# DiffGR: Detecting Differentially Interacting Genomic Regions from Hi-C Contact Maps

**DOI:** 10.1093/gpbjnl/qzae028

**Published:** 2024-03-23

**Authors:** Huiling Liu, Wenxiu Ma

**Affiliations:** Department of Statistics, University of California Riverside, Riverside, CA 92521, USA; Department of Statistics, University of California Riverside, Riverside, CA 92521, USA

**Keywords:** Hi-C, Differential analysis, Topologically associating domain, Stratum-adjusted correlation coefficient, Nonparametric method

## Abstract

Recent advances in high-throughput chromosome conformation capture (Hi-C) techniques have allowed us to map genome-wide chromatin interactions and uncover higher-order chromatin structures, thereby shedding light on the principles of genome architecture and functions. However, statistical methods for detecting changes in large-scale chromatin organization such as topologically associating domains (TADs) are still lacking. Here, we proposed a new statistical method, DiffGR, for detecting differentially interacting genomic regions at the TAD level between Hi-C contact maps. We utilized the stratum-adjusted correlation coefficient to measure similarity of local TAD regions. We then developed a nonparametric approach to identify statistically significant changes of genomic interacting regions. Through simulation studies, we demonstrated that DiffGR can robustly and effectively discover differential genomic regions under various conditions. Furthermore, we successfully revealed cell type-specific changes in genomic interacting regions in both human and mouse Hi-C datasets, and illustrated that DiffGR yielded consistent and advantageous results compared with state-of-the-art differential TAD detection methods. The DiffGR R package is published under the GNU General Public License (GPL) ≥ 2 license and is publicly available at https://github.com/wmalab/DiffGR.

## Introduction

Recent developments of chromatin conformation capture (3C)-based techniques — including chromosome conformation capture-on-chip (4C) [1], chromosome conformation capture carbon copy (5C) [2], high-throughput chromosome conformation capture (Hi-C) [3–5], chromatin interaction analysis with paired-end tag sequencing (ChIA-PET) [6], and HiChIP [7] — have allowed high-throughput characterization of pairwise chromatin interactions in the cell nucleus, and provided an unprecedented opportunity to investigate the three-dimensional (3D) chromatin structures and to elucidate their roles in nuclear organization and gene expression regulation. Among these techniques, Hi-C and its variants [8–10] are of particular interest because of their ability to map chromatin interactions at a genome-wide scale.

A Hi-C experiment yields a symmetric contact matrix in which each entry represents the chromatin contact frequency between the corresponding pair of genomic loci. A particularly important characteristic of Hi-C contact matrices is the presence of the topologically associating domains (TADs), which are functional units of chromatin with higher tendency of intra-domain interactions [11]. TADs are largely conserved across cell types and species. Moreover, CTCF and other chromatin binding proteins are enriched at the TAD boundaries, indicating that TAD boundary regions form chromatin loops and play an essential role in gene expression regulation [11,12].

Several computational methods have been developed to detect TADs in Hi-C contact maps. These methods can be categorized into two groups: one-dimensional (1D) statistic-based methods and two-dimensional (2D) contact matrix-based methods [13]. Of these, 1D statistic-based methods often take a sliding window approach along the diagonal of Hi-C contact matrix and compute a 1D statistic for each diagonal bin to detect TADs and/or TAD boundaries. For instance, Dixon et al. [11] introduced a statistic named directionality index (DI) to quantify whether a genomic locus preferentially interacts with upstream or downstream loci and developed a hidden Markov model to call TADs from DIs. Later, Crane et al. [14] proposed a novel TAD detection method, which computes an insulation score (IS) for each genomic bin by aggregating chromatin interactions within a square sliding through the diagonal and then searches for the minima along the IS profile as TAD boundaries. Unlike the 1D statistic-based methods which calculate statistics using local information, the 2D contact matrix-based methods utilize global information on the contact matrix to capture TAD structures. For example, the Armatus algorithm [15] identifies consistent TAD patterns across different resolutions by maximizing a quality scoring function of domain partition using dynamic programming. In addition, Lévy-Leduc et al. [16] proposed a TAD boundary detection method named HiCseg, which performs a 2D block-wise segmentation via a maximum likelihood approach to partition each chromosome into its constituent TADs. Later, Wang et al. [17] introduced a clustering-based TAD calling method CHDF, which optimizes the clusters of the contact matrix by dynamic programming with an objective function combining the sum of squared error and a penalty term in favor of domain regions with higher frequency of interactions. Recently, several review papers have quantitatively compared the performances of the aforementioned TAD calling methods and demonstrated that HiCseg detects a stable number of TADs against changes of sequencing coverage and maintains the highest reproducibility among Hi-C replicates across all resolutions when compared with other TAD calling methods [18–20].

With the fast accumulation of Hi-C datasets, there has been a growing interest in performing differential analysis of Hi-C contact matrices. To date, several computational tools have been developed for comparative Hi-C analysis, but the majority of them focused on the identification of differential chromatin interactions (DCIs), which represent different chromatin looping events between two Hi-C contact maps. In early studies, the most common strategy for DCI detection was to use the fold change values between two Hi-C contact maps. For instance, Wang et al. [21] used a simple fold change strategy to detect the influence of estrogen treatment on chromatin interactions in MCF-7 Hi-C samples. Additionally, Dixon et al. [22] utilized the fold change values of chromatin interactions to train a random forest model to discover the epigenetic signals that were more predictive of changes in interaction frequencies. In addition to these fold change-based approaches, another commonly utilized method for detecting DCIs was the binomial model implemented by the HOMER software [23]. In contrast, in more recent studies, count-based statistical methods, such as edgeR [24] and DESeq [25], have been adopted to identify pairwise chromatin interactions that show significant changes in contact frequencies. Among them, Lun and Smyth [26] presented a tool named diffHic for rigorous detection of differential interactions by leveraging the generalized linear model (negative binomial regression) of edgeR, and demonstrated that edgeR outperformed the binomial model. Later, Stansfield et al. [27] introduced Minus *vs.* Distance (MD) normalization and performed Z-tests to detect statistically significant DCIs. While all these methods assumed independence among pairwise interactions, which holds true only in coarse-resolution Hi-C maps, Djekidel et al. [28] presented a novel method, named FIND, which takes into account the dependency of adjacent loci at finer resolutions. Briefly, FIND utilizes a spatial Poisson process model to detect DCIs that show significant changes in interaction frequencies of both themselves and their neighborhood bins. Lastly, Cook et al. [29] introduced altered chromatin conformation statistics (ACCOST) to identify differential chromatin contacts by extending the DESeq model used in RNA sequencing (RNA-seq) analysis and repurposing the “size factor” to account for the notable genomic distance effect in Hi-C contact matrices.

In the cell nucleus, chromatin is organized at multiple levels, ranging from active and inactive chromosomal compartments and sub-compartments (on a multi-Mb scale) [3,9], TADs (0.5–2 Mb on average) [11], to fine-scale chromatin interacting loops [8,9]. Chromatin structures also exhibit multi-scale differences among different cell types in their compartments, TADs, and chromatin loops. Among these, changes in TAD organizations are of particular interest as TADs are strongly linked to cell type-specific gene expression [11]. For example, Taberlay et al. [30] have shown that genomic rearrangements in cancer cells are partly guided by changes in higher-order chromatin structures, such as TADs. They discovered that some large TADs in normal cells are further segmented into several smaller TADs in cancer cells, and these changes are tightly correlated with oncogene expression levels. Current differential analyses of TAD structures between different cell types and conditions are limited to the detection of TAD boundary changes. Recently, Chen et al. [13] proposed a TAD boundary detection approach named HiCDB, which is constructed based on local measures of relative insulation and multi-scale aggregation. In addition to calling TAD boundaries in single Hi-C sample, HiCDB also provides differential TAD boundary detection using the average values of relative insulation across multiple samples. Later, Cresswell and Dozmorov [31] developed TADCompare, which uses a spectral clustering-derived metric named eigenvector gap to identify differential and consensus TAD boundaries and track TAD boundary changes over time. Lastly, TADreg [32] introduced a versatile regression framework which generalizes the insulation score by estimating the relative insulating effects of genomic loci and adding a sparsity constraint. The TADreg framework was designed for TAD boundary detection, but also allowed differential TAD analysis across various conditions. The HiCDB, TADCompare, and TADreg methods focused on detecting changes in TAD boundaries rather than changes in chromatin organization within TADs. However, differential TAD boundaries do not necessarily indicate differential chromatin conformation within those regions. First, Hi-C contact matrices are often sparse and noisy, which might lead to unstable detection of TAD boundaries. Second, chromatin interactions within a TAD could be strengthened or weakened in another Hi-C sample, which would suggest different patterns of chromatin organization within the same TAD region. Unfortunately, few methods have been developed to detect differential TAD regions instead of boundaries. Recently, the Hi-C preprocessing and analysis tool HiCExplorer [33–35] expanded its functions to capture differential TAD regions by comparing the precomputed TAD regions on the target Hi-C map with the same regions on the control map by accounting for the information in both intra-TAD and inter-TAD regions. However, such comparison was only limited to the precomputed genomic regions in only one of the Hi-C conditions. Thus, appropriate statistical methods for detecting differentially interacting regions by considering TAD regions across both conditions are still lacking.

To tackle this problem, we developed a novel statistical method, DiffGR, for detecting differential genomic regions at TAD level between two Hi-C contact maps. Briefly, DiffGR utilizes the stratum-adjusted correlation coefficient (SCC), which effectively eliminates the genomic distance effect in Hi-C data, to measure the similarity of local genomic regions between two contact matrices. Subsequently, DiffGR applies a nonparametric permutation test on those SCC values to detect genomic regions with statistically significant differential interactions. We demonstrate, through simulation studies and real data analyses, that DiffGR can effectively and robustly identify differentially interacting genomic regions at the TAD level.

## Method

**Figure 1:**
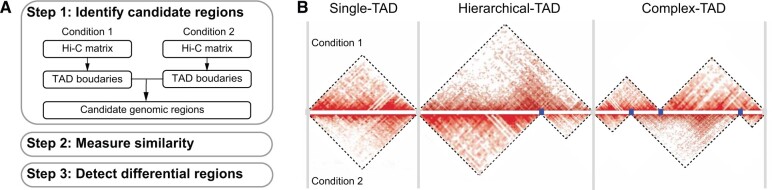
Overview of DiffGR **A**. Workflow of the DiffGR algorithm. **B**. Illustration of three candidate types of differential genomic regions. The gray vertical bars represent the common TAD boundaries between two conditions, which partition the genome into three types of candidate regions. The blue points stand for unique TAD boundaries in only one of the two conditions. Hi-C, high-throughput chromosome conformation capture; TAD, topologically associating domain.

The DiffGR method detects differentially interacting genomic regions in three steps, as shown in **Figure 1**A, and described below in “Identifying candidate genomic regions”, “Measuring similarity of candidate regions between two Hi-C contact maps”, and “Detecting statistically significant differential regions”. In addition, the simulation settings are outlined in “Simulation settings”, and the real data preprocessing and analyses are described in “Real data preprocessing steps”.

### Identifying candidate genomic regions

Suppose that we have two sets of Hi-C data and their corresponding contact frequency matrices as the input. First, we detect the TAD boundaries in each Hi-C data, separately. Specifically, we apply HiCseg [16] to the raw contact matrices and obtain the corresponding TAD boundaries. Note that in this step one can change HiCseg with any other credible TAD caller, such as CDHF [17] or TADreg [32], whose detected TADs satisfy the non-overlapping and continuous properties. We choose HiCseg because it has been shown that HiCseg produces more robust and reliable TAD boundaries than other TAD calling methods [18,20,32]. We next combine the TAD boundaries from both Hi-C contact maps to identify the candidate genomic regions for subsequent analyses. TAD boundaries within two-bin distance are considered to be a common boundary shared by both Hi-C datasets and replaced by the middle bin locus. We then partition the genome into non-overlapping candidate regions using the common TAD boundaries, and categorize these candidate regions into the following three groups: (1) single-TAD candidate regions, (2) hierarchical-TAD candidate regions, and (3) complex-TAD candidate regions, as illustrated in Figure 1B.

We expect different patterns of differential features in these three kinds of candidate genomic regions. As to the differential single-TAD regions, we would expect that strength changes occur in such areas. For differential hierarchical-TAD regions, one large interacting domain could be evidently split into two or more sub-domains, or *vice versa*, boundaries between TADs disappear and thus the corresponding domains merge in one of the contact maps. Lastly, domains might be split, merged, or shifted in a more complicated manner, thereby constructing an entirely new structure, which would be defined as differential complex-TAD regions. Unlike differential single-TAD regions, the differential hierarchical-TAD and complex-TAD regions represent more disruptive changes in the 3D structure of the chromatin.

### Measuring similarity of candidate regions between two Hi-C contact maps

In the second step, we evaluate the similarity of each candidate region between the two samples. Suppose that a candidate genomic region is bounded by two common TAD boundaries shared by both Hi-C maps, and contains k unique TAD boundaries in either one of the two Hi-C maps (shown as blue points in Figure 1B). In the single-TAD candidate region, k=0; in the hierarchical-TAD or complex-TAD candidate regions, k≥1. For each candidate region, we consider all $\left(\begin{array}{c} k\;+\;2\\ \;2 \end{array}\right)$ possible (sub-)TADs, which are separated by any pair of TAD boundaries within that region, as potential differential TADs. For each potential differential TAD, we calculate the SCC [36] rather than the standard Pearson or Spearman correlation coefficients (CCs) to measure the similarity of intra-TAD chromatin interactions between two Hi-C samples. The advantages of using SCC instead of standard CCs are shown in “Supplementary note 1” in File S1.

The SCC metric was introduced by Yang et al. [36] as a measure of similarity and reproducibility between two Hi-C contact matrices. To account for the pronounced distance-dependence effect in Hi-C contact maps, chromatin contacts are first stratified into K strata according to the genomic distances of the contacting locus pairs, and the CCs of contacts within each stratum are calculated between two samples. These stratum-specific CCs are then aggregated to compute the SCC value using a weighted average approach, where the weights are derived from the Cochran–Mantel–Haenszel (CMH) statistic [37]. That is, the SCC ρ is calculated as:

(1) $$\rho =\sum_{k=1}^{K}\left(\frac{N_{k}r_{2k}}{\sum_{k=1}^{K}N_{k}r_{2k}}\right)\rho_{k}$$

where $N_{k}$ is the number of elements in the k-th stratum, $r_{2k}$ is the product of standard deviations of the elements in the k-th stratum of both samples, and $\rho_{k}$ denotes the CC of the k-th stratum between two samples.

The original SCC metric is computed using the intra-chromosomal contact matrices with a predefined genomic distance limit. The resulting value has a range of $\left[-1,1\right]$ and can be interpreted in a way similar to the standard CC. Here, we use SCC as a local similarity measurement to evaluate each potential differential TAD between two Hi-C samples. In the SCC calculation, an upper limit of genomic distance is set to 10 Mb, because TADs are commonly smaller than 10 Mb and distal interactions over a genomic distance larger than 10 Mb are often sparse and highly stochastic. In addition, as the sparsity of Hi-C matrices might affect the precision of SCC values, the locus pairs with zero contact frequencies in both samples are excluded from the calculation.

Hi-C contact maps are often sparse due to sequencing coverage limits and contain various systematic biases. To solve these issues, when preprocessing the Hi-C contact matrices, we first smooth each contact map by a 2D mean filter [36], which substitutes the contact count observed between each bin pair by the average contact count in its neighborhood. This smoothing process improves the contiguity of the TAD regions with elevated contact frequencies, thereby enhancing the domain structures. Next, we utilize the Knight–Ruiz (KR) normalization [38] on the smoothed matrices to remove potential biases.

### Detecting statistically significant differential regions

In the third step, we identify differential genomic regions by first finding differential TADs within these candidate regions. In each candidate genomic region, we calculate the SCC values for all potential differential TADs as described above. Then, we develop a nonparametric permutation test to estimate the *P* values for these local SCC values. Additionally, we propose a quantile regression strategy to speed up the permutation test (see details in “Supplementary methods” in File S1). Finally, we consider a candidate region to be a differentially interacting genomic region, if at least one TAD within that region exhibits a statistically significant difference between the two samples and the size of the largest differential TAD meeting this criterion is greater than one third of the length of the entire candidate region. The longest differential TADs within the detected differentially interacting genomic regions are defined as the noticeable differential areas.

Specifically, we perform the following nonparametric permutation test for each unique TAD size, as the local SCC values are calculated for all potential differential TADs of various sizes.

Suppose that s is a potential differential TAD whose length is $l_{s}$ and the SCC value between two Hi-C samples is $\rho_{s}$. To assess the statistical significance of the observed SCC value $\rho_{s}$, the null distribution of SCC values for TADs of the same size is estimated via the following permutation procedure. To generate a random TAD with length $l_{s}$, we first randomly select $l_{s}$ positions from main diagonal of Hi-C contact matrix, then $l_{s}-1$ position from the first off-diagonal, …, and lastly 1 position from the $\left(l_{s}-1\right)$-th off-diagonal. We subsequently extract contact counts of these randomly selected positions from the two Hi-C contact matrices to construct the permuted TAD pair and calculate its SCC value. We repeat the aforementioned random TAD generation step N times (N=2000) and obtain the corresponding SCC values $\left\{\rho_{i}^{l_{s}}\right\}$, i=1,…,N. Then, the *P* value of the observed SCC value $\rho_{s}$ can be computed as:

(2) $$p_{s}=\frac{\sum_{i=1}^{N}I(\rho_{i}^{l_{s}}\;<\;\rho_{s})}{N}$$

where I(⋅) is the indicator function. Lastly, we compare the *P* values with a pre-defined significance level α (by default α=0.05) to determine differential TADs meeting the significance threshold. Note that the permutation framework accounts for the multiple testing correction using the Benjamini–Hochberg procedure [39].

One potential issue of this permutation framework is the false detection of significantly differential TADs when the two samples are highly similar (*e.g.*, biological replicates from same experiment). This is because the high similarity between biological replicates would lead to high SCC values of the corresponding random TAD patterns. As a result, some non-differential TADs with relatively low SCC values would be falsely detected as differential ones. In order to reduce the number of false positives, we provide an option to filter the *P* values $p_{s}$ by an empirical or automatically calculated threshold. This optional filtering step allows us to pre-specify the meaningful SCC between the two Hi-C datasets that should be reached in order to call a differential TAD truly significant.

(3) $$p_{s}^{\mathrm{adj}}=\left\{\begin{array}{cc} \;0.5 & \;\mathrm{if}\;\;p_{s}<\;\alpha \;\;\mathrm{and}\;\;\rho_{s}>\theta \\ {\;p}_{s} & o\mathrm{therwise} \end{array}\right.$$

The threshold θ can normally be defined as 0.85, which corresponds to a clear margin separating non-replicates from biological/pseudo-replicates in the whole-chromosome similarity comparison between multiple cell lines [40]. Alternatively, θ can be calculated automatically as $\theta =\frac{\rho_{nr}^{l_{s}}+\rho_{br}^{l_{s}}}{2}$, where $\rho_{nr}^{l_{s}}$ represents the mean α quantile of SCCs between non-replicate data and $\rho_{br}^{l_{s}}$ is the mean α quantile of SCCs between their corresponding biological/pseudo-replicate data. Here, we call matrices from different cell lines as non-replicates, matrices from the same cell type as biological replicates, and matrices sampled from pooled biological replicates as pseudo-replicates.

### Simulation settings

To evaluate the performance of the DiffGR method, we conducted a series of simulation experiments by varying the proportion of altered TADs, proportion of TAD alternation, noise level, and sequencing coverage level. Specifically, we utilized the published chromosome 1 contact matrix of K562 cells at 50-kb resolution [9] as the original Hi-C data and simulated the altered Hi-C contact matrices as described below.

#### Single-TAD alternation

Since TADs are conserved genomic patterns and TAD boundaries are relatively stable across cell types and even across species [11], our simulations primarily focused on the scenarios of single-TAD alternations. Suppose that we had an original Hi-C contact matrix M and its identified TAD boundaries. Each of our simulated Hi-C matrices contained two components: the signal matrix S and the noise matrix N, with a certain signal-to-noise ratio.

First, to construct the signal matrix S, we randomly selected a subset of TADs from original contact matrix to serve as the true differential TADs. Then, we replaced a certain portion of contact counts in each selected TAD by randomly sampling contact counts from the corresponding diagonals of the contact matrix. That is, for a chosen contact count located at the bin pair (i,j), we first searched all the bin pairs having the same genomic distance as bin pair (i,j), *i.e.*, $A(v)=\left\{\left(k,l\right):k,l=1,\ldots ,N;l\;\geq \;k,l-k=\left|i-j\right|\right\}$ and randomly selected one position from A(v) and used its corresponding read count to substitute the original value in bin pair (i,j). Second, we simulated the noise matrix N which represents the random ligation events in Hi-C experiments. Briefly, we generated these contacts by randomly choosing two bins, i and j, and adding one to the entry $N_{ij}$ in the noise matrix. The probability of sampling each bin in the bin pair was set proportional to the marginal count of that bin in the original matrix. The sampling process was repeated C times, where C was the total number of contacts in the original Hi-C contact matrix M. The resulting random ligation noise matrix N contained the same number of contacts as the original contact matrix M.

To summarize, we had the following parameters in our single-TAD simulations. (1) Proportion of altered TADs. Using HiCseg, we detected 189 TADs with a mean size of 1.2 Mb in the original K562 chromosome 1 contact matrix (Figure S1). By default, we set the proportion of altered TADs to be 50%, which can vary from 20% to 70%. (2) Proportion of TAD alternation. In the default setting, we substituted all contact counts in the selected TADs by random counts permuted from the matching diagonals in Hi-C maps. To reduce the degree of intra-TAD alternation, we gradually decreased the proportion of randomly substituted intra-TAD contacts from 100% to 10%. (3) Noise level, *i.e.*, the ratio between the noise and signal matrices. The noise level was set to 10% by default, and varied from 1% to 80%.

For each simulation parameter setting, we generated 100 altered Hi-C contact matrices to compare against the original contact matrix. To evaluate the accuracy of the detection results, we used the false detection rate which defines as inaccurate percentage and is computed as $1-\mathrm{Accuracy}=\frac{FP+FN}{N}$, where *FP* denotes the number of falsely detected differential regions, *FN* represents the number of falsely detected non-differential regions, and *N* is the total number of candidate regions being tested.

#### Hierarchical-TAD alternation

In addition to single-TAD alternation, we also simulated the alternation pattern of hierarchical-TADs. We randomly selected 50% of the large TADs whose size was greater than 10 bins in the signal matrix to serve as the true differential TADs. For each of the selected large TAD, we chose a random sub-TAD boundary to split it into two smaller sub-TADs (each with size > 5 bins). We then replaced all inter-sub-TAD contact counts by randomly sampled counts in Hi-C maps. Next, we validated the performance of DiffGR under the hierarchical-TAD condition with respect to different noise levels similar to the single-TAD simulations. Because the complex-TAD condition has complicated TAD boundaries between two samples and occurs less frequently in real data, we did not generate simulation data for this condition.

#### Simulating low-coverage contact matrices

Low sequencing depth of Hi-C experiments would lead to low-coverage and sparse contact matrices, and thus it could potentially affect the performance of the detection of differentially interacting regions. To simulate low-coverage contact matrices, we started with a deep-sequenced Hi-C contact map obtained from human GM12878 cells [9], and down-sampled the contact counts to generate lower-coverage matrices. Specifically, for each non-zero contact count $M_{ij}$ in the original matrix, we assumed that the simulated contact count follows a binomial distribution $M_{ij}^{\prime }∼\mathrm{Binomial}(M_{ij},p)$, where the binomial parameter p={0.2, 0.4, 0.6, 0.8, 1.0} represents the relative coverage level of the down-sampled contact matrix M′. In addition, 10% noise were added to the down-sampled matrices.

### Real data preprocessing steps

In our real data analysis, we used two published Hi-C datasets by Rao et al. (GSE63525) [9] and Dixon et al. (GSE35156) [11] downloaded from Gene Expression Omnibus (GEO). The Rao et al. [9] dataset includes five human cell types: B-lymphoblastoid cells (GM12878), human mammary epithelial cells (HMECs), human umbilical vein endothelial cells (HUVECs), erythrocytic leukemia cells (K562), and normal human epidermal keratinocytes (NHEKs). The GM12878 dataset contains two replicates, which were also pooled together in cell type-specific comparison. The Dixon et al. [11] dataset is from mouse embryonic stem (ES) and cortex cells. Two replicates from mouse ES cells were merged together in cell type-specific comparison. We applied DiffGR to detect differential genomic regions between each pair of cell types at 25-kb, 50-kb, and 100-kb resolutions. Since some of these Hi-C datasets were not deeply sequenced, the local variations introduced by low sequencing coverage made it challenging to capture large domain structures, especially in fine-resolution analyses. Therefore, to enhance the domain structures, all contact matrices were first preprocessed by a 2D mean filter smoothing and then normalized by the KR method to eliminate potential biases. All analyses were performed in parallel using 8 cores on an Intel Core i7-8700K CPU @1.70 GHz with 32 GB of memory allocation. The running time of DiffGR exhibited variation across different resolutions: 3 h for 25-kb, 40 min for 50-kb, and 10 min for 100-kb Hi-C contact maps.

In addition to Hi-C contact maps, chromatin immunoprecipitation sequencing (ChIP-seq) and RNA-seq data from the same cell lines were also included in real data analyses. For ChIP-seq analysis, CTCF and histone modification (H3K4me2, H3K9me3, H3K27ac, and H3K27me3) datasets from five human cell lines in Rao et al. [9] were obtained from the encyclopedia of DNA elements (ENCODE) project [41,42] (https://www.encodeproject.org/). The ChIP-seq files were in Binary Alignment Map (BAM) format. The ChIP-seq peaks were called by MACS2 [43] and stored as narrowpeak/broadpeak Browser Extensible Data (BED) format for the subsequent analyses. In addition, RNA-seq datasets were also obtained from the ENCODE project [42] for human GM12878 and K562 cells (GSE78552 and GSE78625) in read count format, and for mouse ES and cortex cells (GSM723776 and GSM723769) in Fragment Per Kilobase of transcript per Million mapped reads (FPKM) format.

## Results

### DiffGR accurately detected single-TAD differences in simulated datasets

To validate the accuracy and efficiency of our DiffGR method, we first generated pairs of original and simulated Hi-C contact matrices, where a given proportion of TADs in the simulated contact matrices were altered (see Method for details). We used the intra-chromosomal contact matrix of chromosome 1 in K562 cells at 50-kb resolution to serve as the original contact matrix. At the default setting, we altered 50% of the original TADs by completely replacing the intra-TAD contact counts by randomly sampled counts outside the TAD regions. In addition, we added 10% random ligation noise into the altered contact matrices.

**Figure 2:**
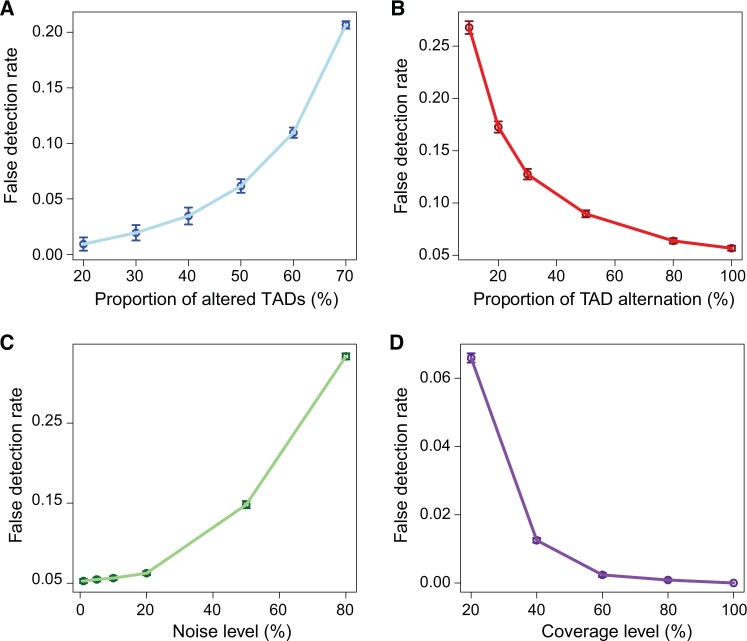
Performance of single-TAD simulations The curves display the mean false detection rates at different levels of proportion of altered TADs (**A**), proportion of TAD alternation (**B**), noise (**C**), and sequencing coverage (**D**). Vertical bars represent 95% confidence intervals.

We first simulated Hi-C matrices with various proportions of altered TADs (20%, 30%, 40%, 50%, 60%, and 70%). With each proportion setting, we completely mutated the intra-TAD counts and added 10% noise, and repeated this simulation procedure 100 times. As expected, the performance of the DiffGR method depended on the proportion of altered TADs. As shown in **Figure 2**A and Table S1, when the proportion of altered TADs changed from 20% to 70%, the false detection rate increased from 0.01 to 0.21. One possible explanation of this observed trend is that when the majority of TADs are altered, the large differences between the original and altered matrices would affect the permutation test and therefore lead to inaccurate detection. However, differential TADs rarely exist in large proportion in real data. The false detection rates of our method remained below 0.07 when the proportion of altered TADs was smaller than or equal to 50%, which demonstrates that our method can accurately and reliably detect single-TAD differences under these conditions.

In the default simulation setting, we completely altered the selected TADs by substituting all intra-TAD contact counts by randomly sampled counts from the matching diagonals outside the TADs. To investigate the influence of the degree of TAD alternation on the DiffGR performance, we generated a series of simulated contact matrices, in which half of original TADs were altered and the proportion of intra-TAD alternation varied from 10%, to 20%, 30%, 50%, 80%, and 100%. In theory, TADs with higher degrees of alternation are easier to identify, whereas TADs with minor changes remain difficult to be detected. As illustrated in Figure 2B and Table S2, the performance of DiffGR improved resulting in higher accuracy as the percentage of randomly substituted counts in altered TADs increased. Even with the most challenging case where only 10% of the intra-TAD counts were altered, the accuracy of our method was 0.73, suggesting that DiffGR can effectively detect subtle TAD differences.

### DiffGR performed stably against changes in noise and coverage levels

Next, we sought to evaluate the robustness of our method under various noise levels and sequencing coverage conditions.

In the earlier simulations, we added 10% noise to the simulated differential contact matrices. To evaluate the performance of our method under different noise levels, we fixed the proportion of altered TADs at 50% and the proportion of intra-TAD alternation at 100%, and simulated the differential contact matrices with a wide range of noise levels (1%, 5%, 10%, 20%, 50%, and 80%). Intuitively, a good detection method should easily discover the differential regions in the less noisy matrices, and it becomes more challenging to detect the differential regions in the noisier cases. Our results demonstrate that DiffGR is able to correctly rank the simulated datasets. We observed a monotonic increasing trend of the false detection rate and a decreasing tendency of other precision measures as the noise levels raised (Figure 2C; Table S3). With moderate noise levels that were not greater than 20%, the accuracy of DiffGR remained above 0.93, indicating that our method can correctly detect differential TAD regions in such noisy cases.

The sequencing coverage of the Hi-C contact maps is another major factor that could affect the performance of our method. Considering two Hi-C replicates that have the same underlying TAD structures but different sequencing coverage levels, we questioned whether our DiffGR method can correctly categorize them as non-differential. In other words, we intended to estimate the false positive rates caused by low-coverage and sparse Hi-C data. To directly investigate the influence of the sequencing coverage on the detection of differential regions, we utilized the GM12878 chromosome 1 contact matrix as the original matrix, and generated a series of down-sampled contact matrices with lower coverage levels (20%, 40%, 60%, 80%, and 100%). The results showed that the average false detection rates remained below 0.05 for most coverage levels, except for the lowest coverage level of 20% (Figure 2D; Table S4), demonstrating the robustness of our DiffGR method under low-coverage conditions.

### DiffGR successfully detected hierarchical-TAD changes

In addition to single-TAD differences, hierarchical-TAD changes also exist in some genomic regions between different cell types. In these regions, one of the Hi-C contact maps exhibits a single dominant TAD structure, while the other Hi-C contact map presents two or more sub-TADs separated by additional boundaries in between. Hierarchical-TADs are computationally challenging to detect. Although the two Hi-C maps have different TAD boundaries, the chromatin interaction patterns within the sub-TADs could be very similar. Consequently, the CCs for the strata with small genomic distances might still remain high between two contact maps. In addition, as the genomic distance increases, the weight of the corresponding stratum in the SCC calculation gradually declines. As a result, the SCC values are primarily contributed by CC values from strata with smaller genomic distances, which makes it difficult to detect differential regions in the hierarchical-TAD cases.

**Figure 3:**
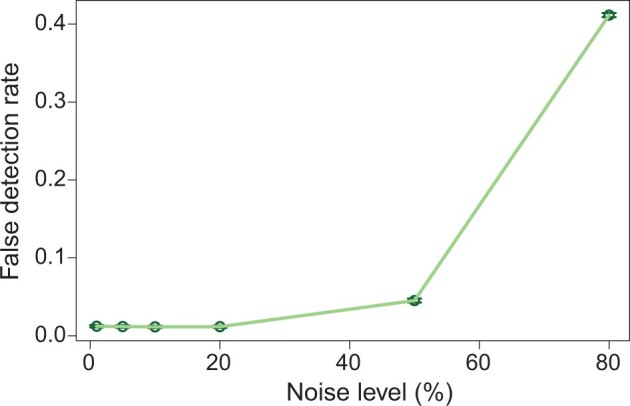
Performance of hierarchical-TAD simulations The curve shows the mean false detection rates at various noise levels. Vertical bars represent 95% confidence intervals.

To evaluate the performance of DiffGR in this more challenging situation, we simulated contact matrices containing hierarchical-TAD structures with respect to varying noise levels (see Method for details), and then computed the false detection rate in a similar manner as in the single-TAD simulations. As demonstrated in **Figure 3** and Table S5, the trend of the false detection rates and other measure statistics across various noise levels under the hierarchical-TAD setting was similar to the pattern observed in the single-TAD case (Figure 2C; Table S3). Furthermore, the false detection rates remained lower than 0.05 when the noise level was within 50%. Taken together, these results indicate that DiffGR can reliably detect the differentially interacting genomic regions with hierarchical-TAD patterns.

### DiffGR revealed cell type-specific genomic interacting regions

Besides validating our method on simulated datasets, we further applied DiffGR to detect cell type-specific differences in five human cell types (GM12878, HMEC, HUVEC, K562, and NHEK) [9] and in two mouse cell types (ES and cortex cells) [11]. In total, we conducted two comparisons between biological replicates in human GM12878 and mouse ES cells, and eleven pairwise comparisons between different cell types (ten pairs among five human cell types and one pair between two mouse cell types). In each pairwise comparison, we first applied HiCseg to identify TAD boundaries from the 50-kb contact matrix for each data and then partitioned the genome into three types of candidate regions: single-TAD candidate regions, hierarchical-TAD candidate regions, and complex-TAD candidate regions. Statistically significant differential genomic regions were identified between each comparison with false discovery rate (FDR) cutoff of 0.05.

We first sought to evaluate the performance of our method on biological replicates of Hi-C data. Previous studies have shown the high degree of similarity between biological replicates and dominant consistence between TAD boundaries in replicate data [9,11,40]. For the comparison between human GM12878 replicates, consistent with our expectations, the majority (89.55%) of the 2325 candidate genomic regions across the genome belonged to single-TAD type, and very few (2.45%) candidate genomic regions were detected as differential by our method (Figure S2). Specifically, only 1.97% of single-TADs were identified as differential, whereas 6.17% and 4.94% were detected as differential in hierarchical-TAD and complex-TAD cases, respectively. Similar results were also witnessed in the comparison between replicates in mouse ES cells: 83.42% candidate genomic regions were classified as single-TAD type and few (6.02%) were identified as differential (Table S6). Overall, our DiffGR results confirmed that these biological replicates displayed highly consistent chromatin structures with minor biological variations.

**Figure 4:**
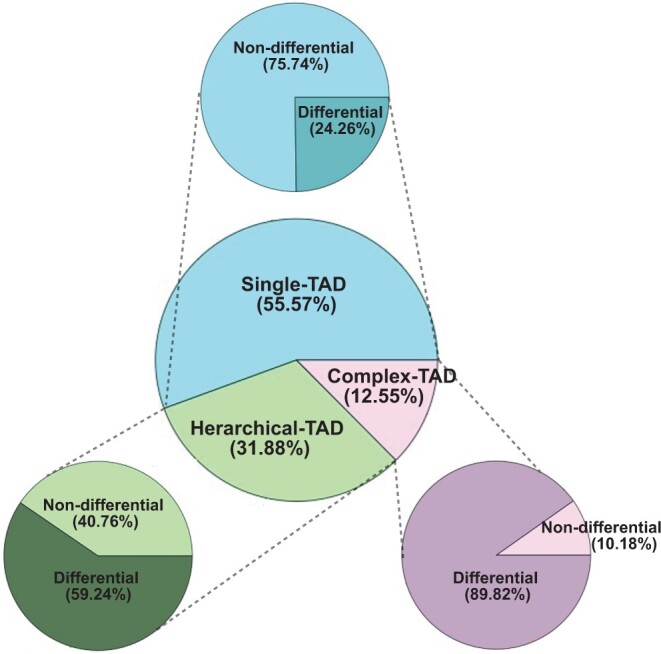
Pie charts of DiffGR results obtained from human Hi-C datasets The DiffGR results from the ten pairwise comparisons among five human cell types (GM12878, HMEC, HUVEC, K562, and NHEK) [9] using the TAD caller HiCseg are summarized. The center pie chart presents the proportions of three categories of candidate regions. The three outer pie charts display the proportions of DiffGR-detected differential genomic regions, one for each candidate category. HMEC, human mammary epithelial cell; HUVEC, human umbilical vein endothelial cell; NHEK, normal human epidermal keratinocyte.

Next, we applied DiffGR to detect cell type-specific differences. As illustrated in **Figure 4**, for the 10 pairwise comparisons among human cell types, 55.57% of all candidate genomic regions belonged to the single-TAD category (consistent with previous observations indicating that TAD boundaries are stable across cell types [11]), 31.88% to the hierarchical-TAD category, and 12.55% to the complex-TAD category. Our DiffGR analyses showed that only 24.26% of the single-TAD candidate regions showed statistically significant differences between two samples; 59.24% of the hierarchical-TAD candidate regions were determined to be differential; while the differential proportion of the complex-TAD category was as high as 89.82%. The differential results were largely consistent when the default TAD caller was changed from HiCseg to CHDF or TADreg, demonstrating the stability of the DiffGR algorithm over different TAD callers (“Supplementary note 2” in File S1). In addition, we found that the proportion of detected differential regions varied largely across chromosomes, ranging from 14% to 76% (Figure S3). For the comparison between mouse ES and cortex cells, 20.22% of the candidate genomic regions in the single-TAD category were identified as differential, while the proportion increased to 75.94% in the complex-TAD category (Table S7). These observations indicate that candidate genomic regions with more distinct patterns of TAD boundaries are more likely to be detected as differential between two Hi-C samples.

In addition to partitioning the genome at 50-kb resolution, we also performed differential analyses on the five human Hi-C datasets at 25-kb and 100-kb resolutions, separately. We calculated the overlapping rate (that is, the proportion of the genome that was classified into the same differential or non-differential status) between different resolutions. Overall, we observed a high consistency between the detected differential regions across different resolutions, where the overlapping rate was 0.8207 between the detection results at 50-kb and 100-kb resolutions, 0.8956 between those at 25-kb and 50-kb resolutions, and 0.7712 between those at 25-kb and 100-kb resolutions. These results demonstrate that DiffGR can robustly and consistently detect cell type-specific differential genomic regions across various resolutions.

### Changes in CTCF and histone modification patterns were consistent with DiffGR detection results

As there is no ground truth of differential chromatin interacting regions in real data, we sought to evaluate the performance of our method by investigating the association between the changes in 1D epigenomic features and 3D genomic interaction regions. The chromatin architectural protein CTCF plays an essential role in establishing higher-order chromatin structures such as TADs. In addition, it has been shown that transcription factors and histone marks are enriched or depleted at TAD boundaries and are associated with active enhancers, promoters, and transcribed genes [11,12]. Therefore, we expected that differential bindings of transcription factors such as CTCF and histone modifications would be more likely located in differential genomic interacting regions.

To test this hypothesis, we then utilized the ChIP-seq datasets of CTCF and histone modifications from the ENCODE project [41]. For each ChIP-seq dataset, we called the peaks via MACS2 [43] and detected differential peaks by DESeq2 [25]. Then, we calculated the proportions of differential peaks that were located in DiffGR-detected differential genomic regions at 100-kb, 50-kb, and 25-kb resolutions. Further, we checked the significance of differential peak enrichment by randomly selecting a bundle of peaks (where the peak number is the same as the number of differential peaks detected by DESeq2) with 2000 times and calculating their corresponding percentages located in differential genomic regions to estimate the *P* values.

**Table 1** summarizes the ChIP-seq analyses on the DiffGR detection results obtained from five human Hi-C datasets [9]. Overall, DiffGR-detected differential genomic regions were supported by 1D epigenomic features. In particular, we observed that the agreement between the changes in ChIP-seq signals and chromatin structures was improved in finer-resolution analyses. As shown in Table 1, 52.48% of the differential CTCF peaks appeared in DiffGR-detected differential genomic regions at 100-kb resolution; whereas in the results at 25-kb resolution, 74.85% of differential CTCF peaks were located in differential genomic regions. In addition, the histone modification datasets showed similar results concordant with the detection results of differentially interacting regions in Hi-C contact maps. At 25-kb resolution, the majority (>70%) of differential histone peaks showed significant consistency with differentially interacting regions for all four histone modification datasets, including H3K4me2, H3K9me3, H3K27ac, and H3K27me3. Collectively, these results indicate that the changes in CTCF bindings and histone modifications are in good agreement with the differences in genomic interacting regions. Furthermore, at finer resolution DiffGR produces more accurate identification of differentially interacting genomic regions in higher agreement with the CTCF and histone modification data.

**Table 1:** Agreements between ChIP-seq data and DiffGR-detected differential genomic regions in human Hi-C datasets

	100-kb resolution	50-kb resolution	25-kb resolution
CTCF	52.48%*	66.15%**	74.85%*
H3K4me2	64.13%*	77.81%***	81.05%***
H3K9me3	51.78%*	65.05%***	72.05%***
H3K27ac	69.98%*	76.95%***	82.56%***
H3K27me3	58.82%*	74.70%***	83.00%***

*Note*: The proportions of differential ChIP-seq peaks located in DiffGR-detected differential genomic regions at 100-kb, 50-kb, and 25-kb resolutions were presented. *, *P* < 0.05; **, *P* < 0.01; ***, *P* < 0.001. ChIP-seq, chromatin immunoprecipitation sequencing; Hi-C, high-throughput chromosome conformation capture.

### Differential RNA-seq analysis results were consistent with DiffGR detection

In addition to investigating the changes in 1D epigenomic features, we further studied the relationship between quantitative changes in gene expression levels and 3D genomic interaction regions. Previous studies have shown that topological changes of 3D genome organization have a large effect on the cross-talk between enhancers and promoters, and therefore can alter gene expression [9,22]. Thus, we expected to observe an enrichment of differential expressed genes in DiffGR-detected differential genomic regions.

To evaluate this assumption, we first detected significant changes in gene expression levels between human GM12878 and K562 cells using DESeq2 [25] and those between mouse ES and cortex cells using Ballgown [44]. Then, we calculated the percentage of differentially expressed genes (DEGs) that were located inside the DiffGR-identified differential genomic regions. To calculate the enrichment of DEGs, we randomly chose a set of genes, whose number is equivalent to the number of the DESeq2-detected DEGs, with 200 times, computed their corresponding proportions located in differential genomic regions, and then performed *t*-test for comparison. In summary, a total number of 8781 DEGs were detected between human GM12878 and K562 cells, and 79.54% of them were located in DiffGR-detected differential genomic regions (*P*$=3.72\;\times \;10^{-5}$, permutation test); whereas 2124 DEGs were identified between mouse ES and cortex cells, and 61.66% were within DiffGR-detected differential genomic regions (*P*$<\;2.2\;\times \;10^{-16}$, permutation test). Taken together, these results demonstrate that the changes of gene expression in RNA-seq data are highly consistent with the DiffGR detection results.

To further explore the potential functional roles of the genes located in differential genomic regions detected by DiffGR, we performed Gene Ontology (GO) enrichment analysis on those genes using the Database for Annotation, Visualization and Integrated Discovery (DAVID) [45]. As shown in **Table 2**, we observed a high enrichment of GO terms related to the immune responses, which is consistent with the immunological nature of GM12878 B-lymphoblastoid cells.

**Table 2:** Functional enrichment of genes located in differential genomic regions between GM12878 and K562

GO term	FDR
GO:0046649 lymphocyte activation	2.7E–11
GO:0002376 immune system process	4.6E–11
GO:0002520 immune system development	2.3E–9
GO:0070663 regulation of leukocyte proliferation	5.2E–7
GO:0042113 B cell activation	7.7E–7
GO:0030183 B cell differentiation	2.2E–5

*Note*: Genes located within differential genomic regions at 25-kb resolution were utilized in GO enrichment analysis. Test results were reported by DAVID [45]. GO, Gene Ontology; FDR, false discovery rate; DAVID, Database for Annotation, Visualization and Integrated Discovery.

### DiffGR detection was supported by DCIs

**Figure 5:**
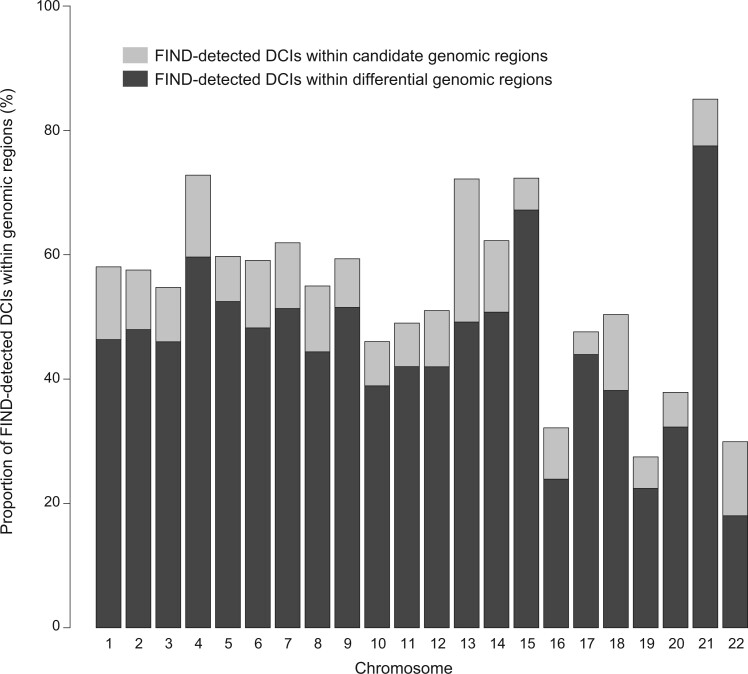
Comparison between FIND and DiffGR Bar chart showing the proportions of FIND-detected DCIs located in candidate genomic regions (light gray) and differential genomic regions (dark gray) for all autosomes between GM12878 and K562. DCI, differential chromatin interaction.

Several Hi-C comparative studies have demonstrated that the majority of the chromatin structural changes tend to couple with the formation/disappearance of TADs [9,22], implying that changes in Hi-C interaction counts are likely to be observed within genomic regions at the TAD level. Hence, we checked DCIs between GM12878 and K562 cells at 50-kb resolution by FIND [28], and compared FIND results with our DiffGR results. As shown in **Figure 5**, the percentages of DCIs detected by FIND located within candidate genomic regions were dominant in the majority of chromosomes (with 55.43% across the whole genome). In addition, 82.80% of the DCIs located in candidate genomic regions were classified into differential regions, demonstrating that DiffGR effectively detects the regions with significant changes in chromatin contacts.

### Performance comparison with state-of-the-art differential TAD detection tools

To further investigate the performance of DiffGR, we compared the DiffGR results with three differential TAD boundary detection methods (HiCDB [13], TADCompare [31], and TADreg [32]) on both simulated and real data. In the simulation part, we compared all four methods using the synthetic data under the default setting (proportion of altered TADs = 50%, proportion of TAD alternation = 100%, and noise level = 10%), and calculated corresponding sensitivities and specificities. As shown in **Table 3**, the sensitivities of the three differential TAD boundary detection methods are all above 70% and comparable to the sensitivity of DiffGR, while the specificities of the three differential TAD boundary detection methods are relatively low. These results demonstrate that HiCDB, TADCompare, and TADreg can accurately identify most differential TAD boundaries within differential regions, but falsely detect many non-differential boundaries within differential regions. We would like to point out that the simulation process was designed to check the robustness of DiffGR detection results by generating random structures within the predefined differential areas and keeping the original chromatin structures within non-differential regions. Therefore, differential TAD boundaries are expected to appear within differential regions, while non-differential TAD boundaries may be located either within or outside the non-differential regions. As a result, the performance on specificities of the differential TAD boundary detection tools was affected.

**Table 3:** Performance comparison of DiffGR and three differential TAD boundary detection tools on simulated data

	Sensitivity		Specificity	
	Mean	SD	Mean	SD
DiffGR	0.8871	0.0239	0.9904	0.0030
HiCDB	0.7758	0.0193	0.0630	0.0415
TADCompare	0.9568	0.0113	0.3128	0.0284
TADreg	0.7762	0.0660	0.3124	0.0303

*Note*: The definitions of the evaluation metrics sensitivity and specificity are explained in “Supplementary methods” in File S1. SD, standard deviation; TAD, topologically associating domain.

Next, we compared the DiffGR results with three differential TAD boundary detection methods on the five human Hi-C datasets by Rao et al. [9] and the two mouse datasets by Dixon et al. [11] at 50-kb resolution. Overall, the differential TAD boundaries identified by HiCDB, TADCompare, and TADreg were highly concordant with DiffGR-detected differentially interacting genomic regions. Notably, 73.86% of the HiCDB-detected, 76.25% of the TADCompare-detected, and 71.90% of the TADreg-detected differential TAD boundaries displayed consistent results with our DiffGR detection in the human datasets. In addition, highly concordant rates were also witnessed in the mouse datasets with 59.56%, 62.01%, and 60.32% consistency rates with HiCDB, TADCompare, and TADreg, respectively.

Furthermore, we compared DiffGR with HiCExplorer [33–35], the only available tool for differential TAD region detection, on the five human Hi-C datasets by Rao et al. [9] at 50-kb resolution. We observed that 60.62% of the 2877 HiCExplorer-identified differential regions overlapped with DiffGR-detected differential regions. To better compare the detection results of DiffGR and HiCExplorer, we then computed the concordant rates between HiCExplorer-detected differential regions and differential ChIP-seq peaks. As shown in **Table 4**, in comparison with DiffGR results, we observed a higher proportion of differential ChIP-seq peaks located in HiCExplorer-detected differential regions, but most of the enrichment are not statistically significant. These results indicate that HiCExplorer identifies a great amount of differential regions; however, some of its detected regions are not significant different based on 1D epigenomic evidence. Further, we investigated the advantages of DiffGR and HiCexplorer over TADCompare, and found that TADCompare-detected differential TAD boundaries within DiffGR-detected differential regions were located closer to the differential ChIP-seq peaks of CTCF and other histone modifications than those outside differential regions, while some disagreements were found in HiCExplorer-detected differential regions (“Supplementary note 3” in File S1). Collectively, these results indicate that DiffGR-detected differential genomic regions have a better agreement with 1D epigenomic features than HiCExplorer-detected differential regions.

**Table 4:** Agreements between ChIP-seq data and differential genomic regions detected by DiffGR and HiCExplorer

	DiffGR	HiCExplorer
CTCF	66.15%**	86.48%
H3K4me2	77.81%***	86.11%
H3K9me3	65.05%***	86.96%***
H3K27ac	76.95%***	86.10%
H3K27me3	74.70%***	85.21%

*Note*: The proportions of differential ChIP-seq peaks located in differential genomic regions detected by DiffGR and HiCExplorer at 50-kb resolution were presented. *, *P* < 0.05; **, *P* < 0.01; ***, *P* < 0.001.

### An example of differentially interacting genomic regions between GM12878 and K562

**Figure 6:**
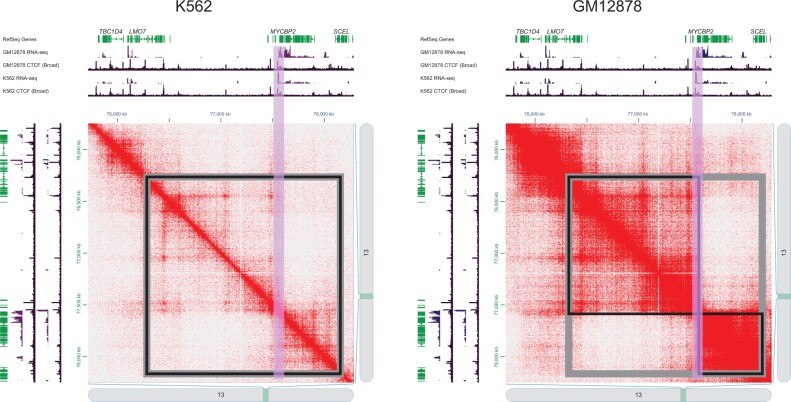
An example of DiffGR-detected differential regions between GM12878 and K562 Hi-C contact maps of the K562 and GM12878 cells at chr13:74,500,000–79,000,000 bp are displayed. The differential genomic region (chr13:75,850,000–78,300,000 bp, with SCC = 0.6551 and *P* = 0.0334) is shown in gray squares, and the TADs for each cell type are shown in black squares. The differential CTCF peak region is highlighted by the purple bars. RNA-seq, RNA sequencing.

**Figure 6** illustrates a differential genomic region (chr13:75,850,000–78,300,000 bp) identified by DiffGR between human GM12878 and K562 cells, which is a hierarchical-TAD region showing a larger TAD in K562 cells and two sub-TADs in GM12878 cells. We observed that the sub-TAD boundary was located inside the MYC binding protein2 (*MYCBP2*) gene which encodes an E3 ubiquitin-protein ligase. Reduced expression of the *MYCBP2* gene has previously been observed in leukemia patients, which has been revealed that CK2 inhibitor takes the anti-leukemia effect through Ikaros-mediated regulation on *MYCBP2* expression in high-risk leukemia [46]. Similarly, we observed a significant differential binding of CTCF at the sub-TAD boundary and an obvious loss of expression in K562 cells (immortalized myelogenous leukemia cells) compared to GM12878 cells (B-lymphocyte cells), which is consistent with the reported clinical association of *MYCBP2* low expression with acute leukemia [46].

## Discussion and conclusion

With the fast accumulation of Hi-C datasets, there has been a dramatically increasing interest in comparative analysis of Hi-C contact maps. However, most existing methods for comparative Hi-C analyses focused on the identification of differential chromatin interactions, while few studies addressed the detection of differential chromatin organization at the TAD scale. To tackle this problem, we developed a novel method, DiffGR, for calling differentially interacting genomic regions between two Hi-C contact maps. Taking genomic distance features of Hi-C data into consideration, our algorithm utilized the SCC metric instead of the standard Pearson CC to measure the similarity of local genomic regions between Hi-C contact maps. Furthermore, we proposed a nonparametric permutation test to assess the statistical significance of the local SCC values. In contrast to the parametric approaches that were used by most Hi-C data analysis methods, our nonparametric approach does not have a set of predefined assumptions about the nature of the null distribution and, therefore, is more robust and can be applied to more diverse data from real cases. Additionally, we utilized a nonparametric smoothing spline regression to speed up the permutation test and showed that the speed-up algorithm can steadily produce consistent outputs. Through empirical evaluations, we have demonstrated that DiffGR can effectively discover differential regions in both simulated data and real Hi-C data from different cell types. That is, DiffGR produced robust and stable detection results under various noise and coverage levels in simulated data; DiffGR detection results in real data were effectively validated by the ChIP-seq and RNA-seq data; DiffGR produced consistent and advantageous results compared with state-of-the-art differential TAD boundary/region detection tools. To summarize, DiffGR provides a statistically rigorous method for the detection of differentially interacting genomic regions in Hi-C contact maps from different cells and conditions, and therefore would facilitate the investigation of their biological functions.

We envision a few possible extensions and future directions based on this work. First, our method performs pairwise comparison between Hi-C contact maps. One potential future direction is to design a more general statistical framework for differential analyses among three or more samples. Then, we could further assign the differentially interacting genomic regions to cell type-specific or condition-specific changing areas. Second, we currently pool biological replicates together in our analyses. Extending DiffGR to incorporate multiple biological replicates to detect reproducible differences would enhance the reliability of the detection results. Third, in our algorithm, we use the shared TAD boundaries between two samples to segment the genome into candidate genomic regions and then detect differential regions. Recently, notion of TADs being highly conserved across different cell types has been challenged [47,48], and there is an increasing recognition of the potential ambiguity in defining TAD boundaries when using different TAD calling methods [49]. Therefore, a more general approach to define and classify the candidate genomic regions would be beneficial to better characterize the variability of chromatin interactions between different conditions. Lastly, our method is specifically designed for bulk Hi-C data. Given the high sparsity and variability of single-cell Hi-C contact matrices, identifying differential genomic regions at single-cell level remains a significant challenge.

## Code availability

The DiffGR R package is publicly available at https://github.com/wmalab/DiffGR under the GNU General Public License (GPL) ≥ 2 license. The source code is also available at BioCode (https://ngdc.cncb.ac.cn/biocode/tools/BT007313).

## CRediT author statement

**Huiling Liu:** Conceptualization, Methodology, Software, Formal analysis, Writing – original draft. **Wenxiu Ma:** Conceptualization, Supervision, Writing – review & editing, Funding acquisition. Both authors have read and approved the final manuscript.

## Supplementary material

Supplementary material is available at *Genomics, Proteomics & Bioinformatics* online (https://doi.org/10.1093/gpbjnl/qzae028).

## Competing interests

Both authors have declared no competing interests.

## Supplementary Material

qzae028_Supplementary_Data
