# Supplementary material for: DiffGR: Detecting Differentially Interacting Genomic Regions from Hi-C Contact Maps
Source: Genomics Proteomics Bioinformatics. 2024 Mar 23;22(2):qzae028. doi: 10.1093/gpbjnl/qzae028 (PMC12016564; doi:10.1093/gpbjnl/qzae028)
Supplement: qzae028_Supplementary_Data [file qzae028_supplementary_data.zip › sup.docx]

**File S1 Supplementary methods and notes**

**Supplementary methods**

### **Speed-up algorithm**

In the third step of the DiffGR algorithm, we perform permutation test to compute $P$-values of 164 local stratum-adjusted correlation coefficients (SCCs) for all potential differential topologically associating domains (TADs). To do so, we need to generate $N$ random TAD pairs for each of the unique TAD sizes. However, such a permutation procedure would be very time-consuming, especially for fine-resolution high-throughput chromosome conformation capture (Hi-C) datasets. To speed up the permutation process, we adopt a nonparametric regression approach to estimate the quantiles of the SCC values. As shown in Figure SS1, we can clearly observe that there is a consistent pattern of the critical values (quantiles) of SCCs that exist for different quantiles and in different datasets. When the TAD size is relatively small, the quantile of SCC values increases dramatically with the TAD size; eventually, when the TAD size is large, the quantile of SCC values levels off. One possible explanation for this observed pattern is that small-size TADs contain an insufficient amount of information to produce reliable local SCC values. As a result, the SCCs of randomly generated small TAD pairs are often low, which would result in low quantile values. As the TAD size increases, sufficient interaction information is obtained from the data. Therefore, the corresponding SCC values would be stabilized leading to relatively reliable and steady quantiles.

To speed up the permutation process, we adopt a nonparametric regression strategy to approximate the SCC quantiles (Figure SS1). Specifically, instead of performing the permutation procedure for all unique TAD sizes, we randomly select a subset of $m$ TAD sizes. For each selected TAD size, we generate $N$ random TAD pairs, compute their local SCCs, and identify a series of quantiles ($\alpha_{1}$-th percentile, $\alpha_{2}$-th percentile, …, $\alpha_{J}$-th percentile) of the SCCs accordingly. Therefore, for a particular quantile (for example, the $\alpha_{j}$-th percentile), we would have $m$ quantile values; one for each of the selected TAD sizes. Based on these $m$ data points, we fit a curve between the $\alpha_{j}$-th percentile and the TAD size via a smoothing spline. Following this regression procedure, for any given TAD size, we can predict a series of quantiles of the SCCs which would be utilized to estimate the $P$-values as previously described in Section 2.3 in Methods of the main manuscript.

As to the selection of TAD sizes, we typically choose $m$ to be 25% of the number of unique TAD sizes that are larger than 15 bins. In addition, we also include all TAD sizes from 1 to 15 bins to obtain an accurate fitting at the beginning of the quantile curve.


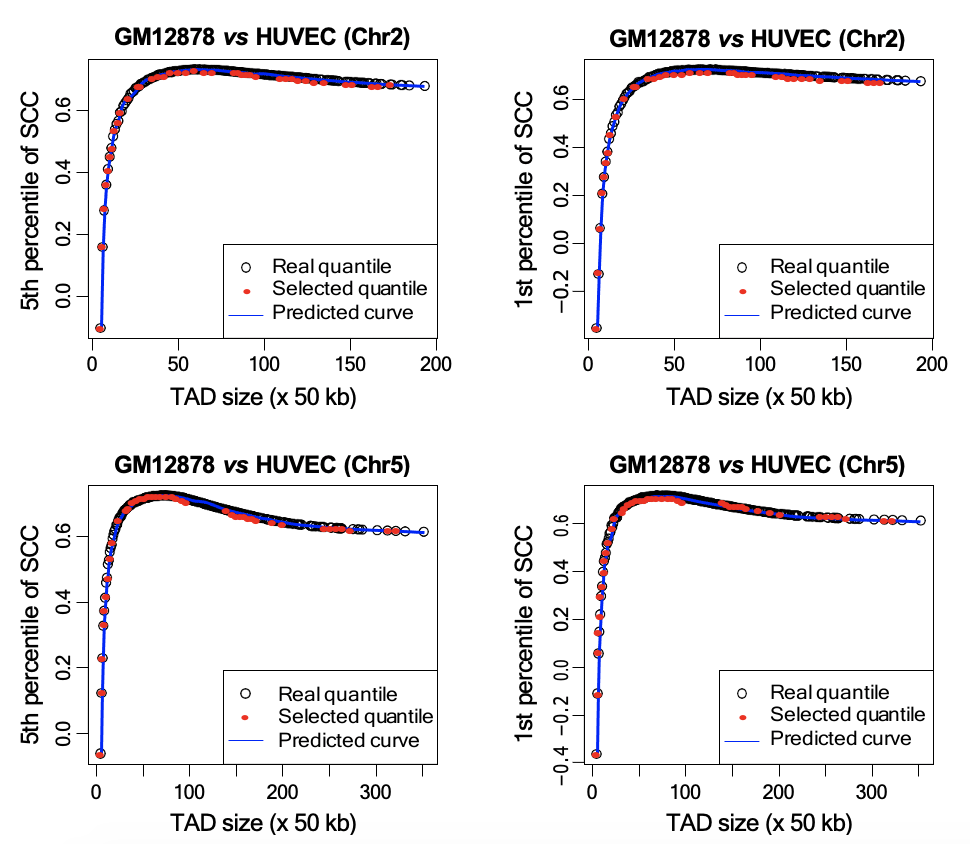


**Figure SS1 Quantiles of local SCC values computed by permutation**

The X-axis indicates the TAD size (bin size = 50 kb), the Y-axis is the corresponding 5th (or 1st) percentile of local SCC values computed from the comparison between GM12878 and HUVEC cells. The open black circles represent real quantile values; the red points denote the randomly selected points by the speed-up algorithm to fit the regression line; the blue line is the predicted quantile curve by a smooth spline. ﻿TADs, topologically associating domains; SCC, stratum-adjusted correlation coefficient; HUVEC, human umbilical vein endothelial cells.

### **Explanation of supplementary tables**

The evaluation statistics measuring the performance of DiffGR on simulated data are listed in Table SS1.

**Table SS1 The illustration of evaluation statistics**

| **Metric** | **Definition** |
| --- | --- |
| TP | True positives |
| FP | False positives |
| TN | True negatives |
| FN | False negatives |
| Sensitivity | TP/(TP+FN) |
| Specificity | TN/(FP+TN) |
| Accuracy | (TP+TN)/(TP+FP+TN+FN) |
| Precision | TP/(TP+FP) |
| F1 score | 2TP/(2TP+FP+FN) |
| MCC | Matthews correlation coefficient: $\frac{TP\times TN-FP\times FN}{\sqrt{\left( TP+FP \right)\left( TP+FN \right)\left( TN+FP \right)\left( TN+FN \right)}}$ |

**Supplementary notes**

### **SCC outperformed Pearson correlation coefficient in measuring the similarity of local TAD regions**

In the proposed DiffGR method, we used SCC to measure the similarity of local TAD regions between two Hi-C contact maps. In addition to SCC, other commonly used similarity measurements for comparing Hi-C contact matrices include Pearson and Spearman correlation coefficients (CCs). The main advantage of SCC over the standard CCs is that SCC explicitly takes the genomic distance effect into consideration, thereby achieving better performance in evaluating the Hi-C contact matrices. Therefore, we expected SCC to serve as a good metric to compare chromatin interacting patterns at local TAD regions.

To validate our choice of the SCC similarity metric, we tested a variation of the DiffGR method that substitutes SCC with the standard Pearson CC, and evaluated its performance using the simulated Hi-C contact matrices with various proportions of altered TADs and across different noise levels. As shown in Figure SS2A and Table SS2, our method utilizing SCC evidently outperformed the alternative version employing Pearson CC in terms of various proportions of altered TADs. For each proportion of altered TADs, the false detection rates based on Pearson CC were significantly higher than those relying on SCC. Moreover, the variations of the false detection rates measured by SCC were much smaller than those obtained by Pearson CC. Therefore, these results demonstrated that SCC is indeed a better similarity metric than Pearson CC in measuring local TAD patterns between Hi-C contact matrices. The advantage of utilizing SCC instead of Pearson CC was also witnessed in simulation results across different noise levels. %Since the counts in the noise matrix are regarded as random ligation events, we expect the simulated data with high noise levels would be fair to compare SCC with Pearson CC. As shown in Figure SS2B, SCC significantly outperformed Pearson CC at all noise levels, demonstrating the advantage of SCC measuring the similarity of intra-TAD chromatin interactions between two Hi-C samples.


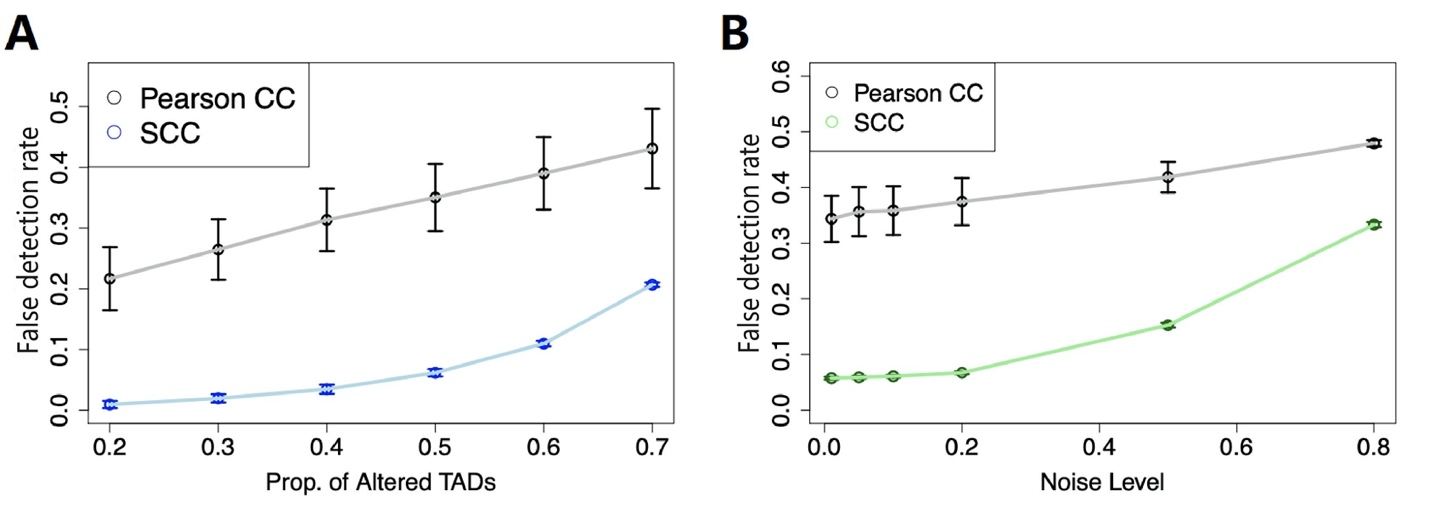


**Figure SS2 Comparison between SCC and Pearson CC**

The curves represent the mean false detection rates at (A) various proportions of altered TADs and (B) various noise levels using either SCC or Pearson CC as the local similarity metric. Vertical bars represent 95% confidence intervals. CC, correlation coefficient; Prop., proportion.

**Table SS2 Evaluation of Pearson correlation coefficient performance on DiffGR detection**

|  | **0.2** | **0.3** | **0.4** | **0.5** | **0.6** | **0.7** |
| --- | --- | --- | --- | --- | --- | --- |
| TP | 7.96 | 5.98 | 1.57 | 0.74 | 0.47 | 0.41 |
| FP | 33.67 | 33.16 | 30.37 | 29.33 | 28.97 | 29.01 |
| TN | 140.13 | 133.04 | 128.23 | 122.07 | 114.83 | 107.19 |
| FN | 7.24 | 16.82 | 28.83 | 36.86 | 44.73 | 52.39 |
| Sensitivity | 0.8095 | 0.7049 | 0.6207 | 0.6079 | 0.6042 | 0.6031 |
| Specificity | 0.8215 | 0.8241 | 0.8388 | 0.8448 | 0.8467 | 0.8465 |
| Accuracy | 0.7835 | 0.7356 | 0.6868 | 0.6498 | 0.6101 | 0.5693 |
| Precision | 0.4321 | 0.4441 | 0.4546 | 0.4300 | 0.4400 | 0.4000 |
| F1 score | 0.2730 | 0.1805 | 0.0933 | 0.0451 | 0.0482 | 0.0062 |
| MCC | 0.8515 | 0.7526 | 0.6817 | 0.6463 | 0.6449 | 0.6194 |

*Note*: The proportion of altered TADs varies from 0.2 to 0.7. The definitions of the evaluation metrics are explained in Table SS1. TADs, topologically associating domains.

### **Comparison of DiffGR results using different TAD callers**

In the DiffGR algorithm, we use HiCseg as the default TAD calling method for identifying the candidate genomic regions. The main reason for choosing HiCseg is that its detection results were shown to outperform most state-of-the-art TAD callers in terms of robustness and reliability [1-3]. Besides HiCseg, other reliable TAD callers, whose detected TADs satisfy the non-overlapping and continuous properties, can also be utilized to classify the candidate genomic regions. Although different TAD callers may produce slightly different candidate genomic regions, we expect that DiffGR is able to identify statistically significant differentially interacting regions in a consistent manner.

To validate the assumption, we identified the TAD boundaries of five human cell lines using two other credible TAD calling methods CHDF [4] and TADreg [3] . CHDF was listed as one of the top-performing TAD boundary detection methods in a review paper by Zufferey and his colleagues [2]. TADreg was shown to outperform existing TAD calling methods by . Compared with the DiffGR results given by HiCseg (Figure 4 ; Table SS3), the DiffGR detection results of candidate genomic regions and differentially interacting regions using CHDF and TADreg are displayed in Tables SS4 and SS5, respectively. Although the proportion and differential rate of each candidate region category varied among HiCseg, CHDF, and TADreg, we observed a common trend in the DiffGR detection results. That is, the majority of candidate genomic regions were classified as single-TADs, followed by hierarchical-TADs and then complex-TADs; significantly higher proportions of differentially interacting regions were detected in candidate genomic regions where more distinct patterns of TAD boundaries appeared (hierarchical-TADs and complex-TAD).

We further calculated the pairwise concordant rates of differential genomic regions among three TAD calling methods. Specifically, we define a DiffGR-detected differential region given by a TAD caller as consistent to the results given by another TAD caller if over one third of the region is identified to be differential in the DiffGR results by the other TAD caller. As shown in Table SS6, 90.22% and 88.24% of the DiffGR-detected differential genomic regions using HiCseg overlapped with the differential detection results using TADreg and CHDF, respectively; the detection consistency between CHDF and TADreg was also above 70%. Taken together, these results demonstrated the stability of differential region detection results over different TAD callers.

**Table SS3 Summary of DiffGR results obtained from human Hi-C datasets using TAD caller HiCseg**

| **Candidate region category** | **Proportion** | **Differential proportion** |
| --- | --- | --- |
| Single-TAD | 55.57% | 24.26% |
| Hierarchical-TAD | 31.88% | 59.24% |
| Complex-TAD | 12.55% | 89.82% |

*Note*: The first column lists three types of candidate genomic regions and the second one displays their corresponding proportions. The third column shows the proportions of DiffGR-detected differential genomic regions in each candidate category. Hi-C, high-throughput chromosome conformation capture.

**Table SS4 Summary of DiffGR results obtained from human Hi-C datasets using TAD caller CHDF**

| **Candidate region category** | **Proportion** | **Differential proportion** |
| --- | --- | --- |
| Single-TAD | 70.95% | 18.74% |
| Hierarchical-TAD | 19.49% | 42.86% |
| Complex-TAD | 9.56% | 73.98% |

*Note*: The first column lists three types of candidate genomic regions and the second one displays their corresponding proportions. The third column shows the proportions of DiffGR-detected differential genomic regions in each candidate category. Hi-C, high-throughput chromosome conformation capture.

**Table SS5 Summary of DiffGR results obtained from human Hi-C datasets using TAD caller TADreg**

| **Candidate region category** | **Proportion** | **Differential proportion** |
| --- | --- | --- |
| Single-TAD | 56.90% | 26.95% |
| Hierarchical-TAD | 32.99% | 59.53% |
| Complex-TAD | 10.11% | 78.31% |

*Note*: The first column lists three types of candidate genomic regions and the second one displays their corresponding proportions. The third column shows the proportions of DiffGR-detected differential genomic regions in each candidate category. Hi-C, high-throughput chromosome conformation capture.

**Table SS6 Concordant rates of DiffGR results obtained from human Hi-C datasets using different TAD callers**

| **Method A** | **Method B** | **Consistency rate (A *vs* B)** | **Consistency rate (A *vs* B)** |
| --- | --- | --- | --- |
| HiCseg | CHDF | 88.24% | 69.81% |
| HiCseg | TADreg | 90.22% | 76.18% |
| CHDF | TADreg | 74.87% | 78.39% |

*Note*: The first and second columns list TAD calling methods A and B. The third column shows the proportion of DiffGR-detected differential regions using TAD-calling method A showing as differential in results obtained by method B. The fourth column displays the proportions of differential regions detected using method B showing as differential in results obtained by method A.

### **DiffGR achieved a better agreement with ChIP-seq data than HiCExplorer in terms of differential results over TADCompare**

Next we sought to assess the agreement between Chromatin Immunoprecipitation Sequencing (ChIP-seq) data and the differential genomic regions detected by DiffGR and HiCExplorer. We first use TADCompare to detect differential TAD boundaries and classified the TADCompare-detected differential boundaries into two groups: those within the detected differential regions (DRs) and those outside the differential regions (NDRs). Then we calculated the log distances of the differential TAD boundaries within/outside differential regions to their nearest differential ChIP-seq peaks of CTCF and histone modifications. From Figure SS3 and Table SS7, we observed that differential TAD boundaries within differential regions were located relatively closer to the differential CTCF peaks than those outside differential regions. Furthermore, the histone modification datasets (including H3K4me2, H3K9me3, H3K27ac, and H3K27me3) showed similar results that were in agreement with the advantageous results of differential TAD boundaries in differentially interacting regions given by DiffGR. However, some disagreements were found in HiCExplorer-detected differential regions with respect to histone modification H3K9me3 and H3K27me3 data. Collectively, these results indicated that DiffGR-detected differential genomic regions had a better agreement with one-dimensional (1D) epigenomic features than HiCExplorer-detected differential regions.

**Table SS7 Distance between differential ChIP-seq peaks and differential TAD boundaries given by TADCompareC**

|  | **DiffGR** | | | | **HiCExplorer** | | |
| --- | --- | --- | --- | --- | --- | --- | --- |
|  | **DR** | **NDR** | **Significance** | **DR** | | **NDR** | **Significance** |
| CTCF | 3.1304 | 3.3281 | *** | 3.1649 | | 3.2961 | *** |
| H3K4me2 | 1.2464 | 1.8164 | *** | 1.3615 | | 1.4886 | *** |
| H3K9me3 | 2.6970 | 3.0610 | *** | 2.7823 | | 2.7839 | 0.4809 |
| H3K27ac | 1.0345 | 1.4337 | *** | 1.1176 | | 1.1900 | *** |
| H3K27me3 | 2.1267 | 2.5603 | *** | 2.2390 | | 2.1709 | 0.9961 |

*Note*: The DR and NDR columns represent the mean log distances of the TADCompare-detected differential TAD boundaries within differential genomic regions (DRs) and non-differential regions (NDRs) to their nearest differential ChIP-seq peaks. The P-values of the sample mean *t*-tests between DR and NDR groups were shown as significant codes: "***" if *P*-value <0.001, "**" if 0.001<*P*-value <0.01,"*" if $0.01< *P*-value <0.05, and the exact *P*-value if P-value >0.05. CHIP-seq, chromatin immunoprecipitation sequencing.


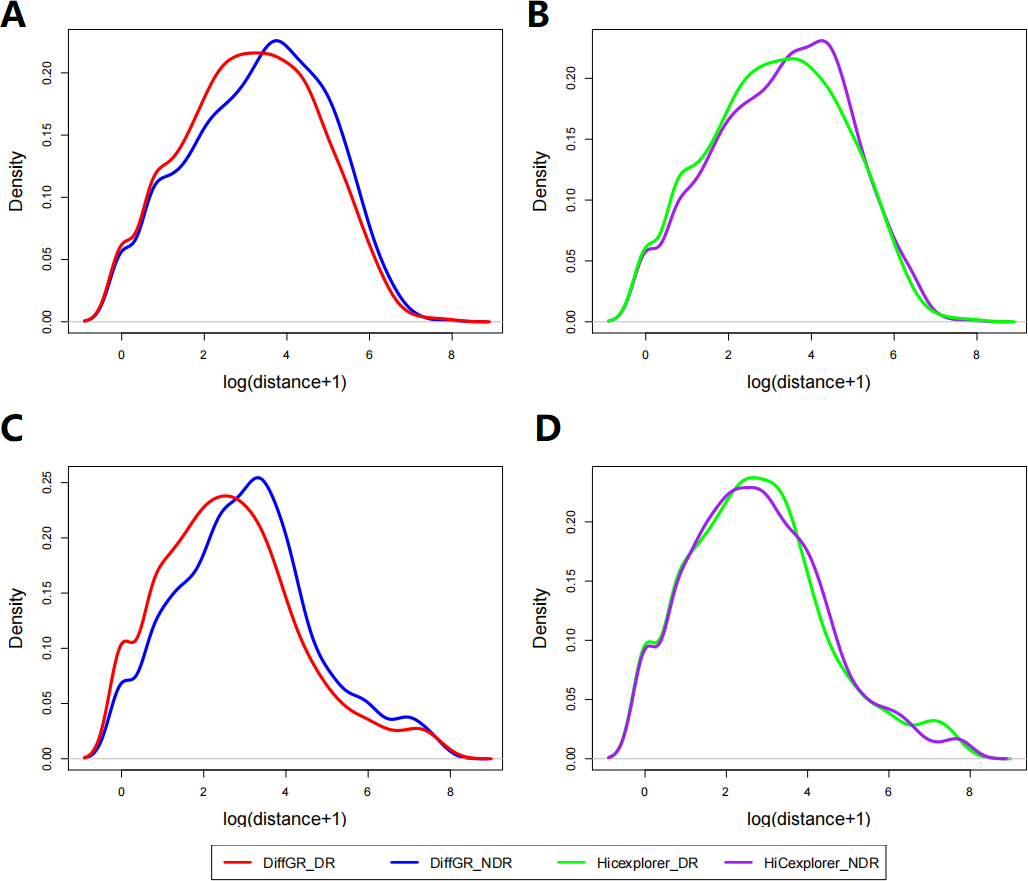


**Figure SS3 Comparison between DiffGR and HiCExplorer in terms of differential results over TADCompare**

Density plots of log distances of TADCompare-detected differential TAD boundaries within differential genomic regions (DRs) and non-differential regions (NDRs) to their nearest differential ChIP-seq peaks. Panels A and B display the density curves of log distance to differential CTCFpeaks; C and D show the density curves of the log distance to differential H3K9me3 peaks. The red and blue curves are for DiffGR-detected DRs and NDRs, respectively. The green and purple curves are for HiCExplorer-detected DRs and NDRs, respectively.

**References**

﻿[1] Forcato M, Nicoletti C, Pal K, Livi CM, Ferrari F, Bicciato S. Comparison of computational

methods for Hi-C data analysis. Nat Methods 2017;14:679–85.

[2] Zufferey M, Tavernari D, Oricchio E, Ciriello G. Comparison of computational methods for the identification of topologically associating domains. Genome Biol 2018;19:217.

[3] Mourad R. TADreg: a versatile regression framework for TAD identification, differential

analysis and rearranged 3D genome prediction. BMC Bioinformatics 2022;23:82.

[4] Wang Y, Li Y, Gao J, Zhang MQ. A novel method to identify topological domains using Hi-C data. Quant Biol 2015;3:81–9.
