# Supplementary material for: DiffGR: Detecting Differentially Interacting Genomic Regions from Hi-C Contact Maps
Source: Genomics Proteomics Bioinformatics. 2024 Mar 23;22(2):qzae028. doi: 10.1093/gpbjnl/qzae028 (PMC12016564; doi:10.1093/gpbjnl/qzae028)
Supplement: qzae028_Supplementary_Data [file qzae028_supplementary_data.zip › Supplementary material captions.docx]

**Supplementary material**

**File S1 Supplementary methods and notes**

**Figure S1 Histogram of TAD size of all HiCseg identified TADs on Chromosome 1 of K562 cells at 50-kb resolution**

**Figure S2 Pie charts of DiffGR results obtained from human GM12878 Hi-C datasets**

**A.** Pie chart presents the proportions of three types of candidate regions. The rest three pie charts display the proportions of detected differential genomic regions in each candidate category in which blue for single-TADs (**B**), green for hierarchical-TADs (**C**), and purple for complex-TADs (**D**).

**Figure S3 Summary of DiffGR-detected differential genomic regions in human Hi-C datasets**

**A.** Histograms of chromosome-wide proportion of differentially interacting genomic regions for all pairwise comparisons between two cell types. **B.** Bar plots of the numbers of candidate regions and detected differential genomic regions per chromosome for all pairwise comparisons between two cell types.

**Table S1 Evaluation of the effect of proportion of altered TADs on DiffGR detection**

**Table S2 Evaluation of the effect of proportion of TAD alternation on DiffGR detection**

**Table S3 Evaluation of the effect of noise level on DiffGR detection**

**Table S4 Evaluation of the effect of coverage level on DiffGR detection**

**Table S5 Evaluation of the effect of hierarchical setting on DiffGR detection**

**Table S6 Summary of DiffGR results obtained from biological replicates of mouse ES cells**

**Table S7 Summary of DiffGR results obtained from mouse ES and cortex cells**
