# Supplementary material for: DiffGR: Detecting Differentially Interacting Genomic Regions from Hi-C Contact Maps
Source: Genomics Proteomics Bioinformatics. 2024 Mar 23;22(2):qzae028. doi: 10.1093/gpbjnl/qzae028 (PMC12016564; doi:10.1093/gpbjnl/qzae028)
Supplement: qzae028_Supplementary_Data [file qzae028_supplementary_data.zip › Table S1.docx]

**Table S1 Evaluation of the effect of proportion of altered TADs on DiffGR detection**

|  | **0.2** | **0.3** | **0.4** | **0.5** | **0.6** | **0.7** |
| --- | --- | --- | --- | --- | --- | --- |
| TP | 36.53 | 53.64 | 69.82 | 82.62 | 92.39 | 92.95 |
| FP | 0.29 | 0.33 | 0.36 | 0.27 | 0.11 | 0.00 |
| TN | 150.71 | 131.67 | 112.64 | 94.73 | 75.89 | 57.00 |
| FN | 1.47 | 3.36 | 6.18 | 11.38 | 20.61 | 39.05 |
| Sensitivity | 0.9613 | 0.9411 | 0.9187 | 0.8789 | 0.8176 | 0.7042 |
| Specificity | 0.9981 | 0.9975 | 0.9968 | 0.9972 | 0.9986 | 1.0000 |
| Accuracy | 0.9907 | 0.9805 | 0.9654 | 0.9384 | 0.8904 | 0.7934 |
| Precision | 0.9924 | 0.994 | 0.9949 | 0.9967 | 0.9988 | 1.0000 |
| F1 score | 0.9764 | 0.9666 | 0.9551 | 0.9339 | 0.8988 | 0.8262 |
| MCC | 0.9709 | 0.9537 | 0.9292 | 0.883 | 0.8011 | 0.6468 |

*Note*: The proportion of altered TADs varies from 0.2 to 0.7. The definitions of the evaluation metrics are explained in “Supplementary methods” in File S1. TP, true positives; FP, false positives; TN, true negatives; FN, false negatives; MCC, Matthews correlation coefficient.
