# Supplementary material for: DiffGR: Detecting Differentially Interacting Genomic Regions from Hi-C Contact Maps
Source: Genomics Proteomics Bioinformatics. 2024 Mar 23;22(2):qzae028. doi: 10.1093/gpbjnl/qzae028 (PMC12016564; doi:10.1093/gpbjnl/qzae028)
Supplement: qzae028_Supplementary_Data [file qzae028_supplementary_data.zip › Table S2.docx]

**Table S2 Evaluation of the effect of proportion of TAD alternation on DiffGR detection**

|  | **0.1** | **0.2** | **0.3** | **0.5** | **0.8** | **1** |
| --- | --- | --- | --- | --- | --- | --- |
| TP | 43.38 | 61.37 | 69.9 | 77.06 | 81.95 | 83.28 |
| FP | 0.00 | 0.00 | 0.00 | 0.00 | 0.00 | 0.00 |
| TN | 95.00 | 95.00 | 95.00 | 95.00 | 95.00 | 95.00 |
| FN | 50.62 | 32.63 | 24.1 | 16.94 | 12.05 | 10.72 |
| Sensitivity | 0.4615 | 0.6529 | 0.7436 | 0.8198 | 0.8718 | 0.8860 |
| Specificity | 1.0000 | 1.0000 | 1.0000 | 1.0000 | 1.0000 | 1.0000 |
| Accuracy | 0.7322 | 0.8274 | 0.8725 | 0.9104 | 0.9362 | 0.9433 |
| Precision | 1.0000 | 1.0000 | 1.0000 | 1.0000 | 1.0000 | 1.0000 |
| F1 score | 0.6291 | 0.7886 | 0.8520 | 0.9006 | 0.9313 | 0.9394 |
| MCC | 0.5485 | 0.6975 | 0.7707 | 0.8344 | 0.8798 | 0.8925 |

*Note*: The proportion of TAD alternation varies from 0.1 to 1.0. The definitions of the evaluation metrics are explained in “Supplementary methods” in File S1.
