# Supplementary material for: DiffGR: Detecting Differentially Interacting Genomic Regions from Hi-C Contact Maps
Source: Genomics Proteomics Bioinformatics. 2024 Mar 23;22(2):qzae028. doi: 10.1093/gpbjnl/qzae028 (PMC12016564; doi:10.1093/gpbjnl/qzae028)
Supplement: qzae028_Supplementary_Data [file qzae028_supplementary_data.zip › Table S3.docx]

**Table S3 Evaluation of the effect of noise level on DiffGR detection**

|  | **0.01** | **0.05** | **0.1** | **0.2** | **0.5** | **0.8** |
| --- | --- | --- | --- | --- | --- | --- |
| TP | 84.06 | 83.75 | 83.39 | 82.23 | 66.04 | 32.20 |
| FP | 0.94 | 0.92 | 0.91 | 0.94 | 0.89 | 1.18 |
| TN | 94.06 | 94.08 | 94.09 | 94.06 | 94.11 | 93.82 |
| FN | 9.94 | 10.25 | 10.61 | 11.77 | 27.96 | 61.80 |
| Sensitivity | 0.8943 | 0.8910 | 0.8871 | 0.8748 | 0.7026 | 0.3426 |
| Specificity | 0.9901 | 0.9903 | 0.9904 | 0.9901 | 0.9906 | 0.9876 |
| Accuracy | 0.9424 | 0.9409 | 0.9390 | 0.9328 | 0.8474 | 0.6668 |
| Precision | 0.9889 | 0.9891 | 0.9892 | 0.9887 | 0.9867 | 0.9652 |
| F1 score | 0.9390 | 0.9373 | 0.9352 | 0.9281 | 0.8199 | 0.5040 |
| MCC | 0.8891 | 0.8863 | 0.8829 | 0.8714 | 0.7251 | 0.4327 |

*Note*: The noise level varies from 0.01 to 0.8. The definitions of the evaluation metrics are explained in “Supplementary methods” in File S1.
