# Supplementary material for: DiffGR: Detecting Differentially Interacting Genomic Regions from Hi-C Contact Maps
Source: Genomics Proteomics Bioinformatics. 2024 Mar 23;22(2):qzae028. doi: 10.1093/gpbjnl/qzae028 (PMC12016564; doi:10.1093/gpbjnl/qzae028)
Supplement: qzae028_Supplementary_Data [file qzae028_supplementary_data.zip › Table S4.docx]

**Table S4 Evaluation of the effect of coverage level on DiffGR detection**

|  | **1** | **0.8** | **0.6** | **0.4** | **0.2** |
| --- | --- | --- | --- | --- | --- |
| TP | 0.00 | 0.00 | 0.00 | 0.00 | 0.00 |
| FP | 0.00 | 0.19 | 0.52 | 2.75 | 14.51 |
| TN | 220.00 | 219.81 | 219.48 | 217.25 | 205.49 |
| FN | 0.00 | 0.00 | 0.00 | 0.00 | 0.00 |
| Sensitivity | 1.0000 | 1.0000 | 1.0000 | 1.0000 | 1.0000 |
| Specificity | 1.0000 | 0.9991 | 0.9976 | 0.9875 | 0.9341 |
| Accuracy | 1.0000 | 0.9991 | 0.9976 | 0.9875 | 0.9341 |
| Precision | 1.0000 | 0.8100 | 0.5634 | 0.0000 | 0.0000 |
| F1 score | 1.0000 | 0.8100 | 0.5634 | 0.0000 | 0.0000 |
| MCC | 1.0000 | 1.0000 | 1.0000 | 1.0000 | 1.0000 |

*Note*: The coverage level varies from 1.0 to 0.2. The definitions of the evaluation metrics are explained in “Supplementary methods” in File S1.
