# Supplementary material for: DiffGR: Detecting Differentially Interacting Genomic Regions from Hi-C Contact Maps
Source: Genomics Proteomics Bioinformatics. 2024 Mar 23;22(2):qzae028. doi: 10.1093/gpbjnl/qzae028 (PMC12016564; doi:10.1093/gpbjnl/qzae028)
Supplement: qzae028_Supplementary_Data [file qzae028_supplementary_data.zip › Table S5.docx]

**Table S5 Evaluation of the effect of hierarchical setting on DiffGR detection**

|  | **0.01** | **0.05** | **0.1** | **0.2** | **0.5** | **0.8** |
| --- | --- | --- | --- | --- | --- | --- |
| TP | 91.67 | 91.75 | 91.82 | 91.78 | 85.58 | 16.71 |
| FP | 0.00 | 0.00 | 0.00 | 0.00 | 0.13 | 0.48 |
| TN | 95.00 | 95.00 | 95.00 | 95.00 | 94.87 | 94.52 |
| FN | 2.33 | 2.25 | 2.18 | 2.22 | 8.42 | 77.29 |
| Sensitivity | 0.9752 | 0.9761 | 0.9768 | 0.9764 | 0.9104 | 0.1778 |
| Specificity | 1.0000 | 1.0000 | 1.0000 | 1.0000 | 0.9986 | 0.9949 |
| Accuracy | 0.9877 | 0.9881 | 0.9885 | 0.9883 | 0.9548 | 0.5885 |
| Precision | 1.0000 | 1.0000 | 1.0000 | 1.0000 | 0.9985 | 0.9725 |
| F1 score | 0.9874 | 0.9878 | 0.9882 | 0.9880 | 0.9522 | 0.2998 |
| MCC | 0.9757 | 0.9766 | 0.9773 | 0.9769 | 0.9133 | 0.2998 |

*Note*: The noise level varies from 0.01 to 0.8. The definitions of the evaluation metrics are explained in “Supplementary methods” in File S1.
