# Supplementary material for: DiffGR: Detecting Differentially Interacting Genomic Regions from Hi-C Contact Maps
Source: Genomics Proteomics Bioinformatics. 2024 Mar 23;22(2):qzae028. doi: 10.1093/gpbjnl/qzae028 (PMC12016564; doi:10.1093/gpbjnl/qzae028)
Supplement: qzae028_Supplementary_Data [file qzae028_supplementary_data.zip › Table S7.docx]

**Table S7 Summary of DiffGR results obtained from mouse ES and cortex cells**

| **Candidate region category** | **Proportion** | **Differential proportion** |
| --- | --- | --- |
| Single TAD | 33.09% | 20.22% |
| Hierarchical TAD | 52.23% | 46.62% |
| Complex TAD | 14.68% | 75.94% |

*Note*: The first column lists three types of candidate genomic regions and the second one displays their corresponding proportions. The third column shows the proportions of DiffGR-detected differential genomic regions in each candidate category.
