# Supplementary material for: DiffGR: Detecting Differentially Interacting Genomic Regions from Hi-C Contact Maps
Source: Genomics Proteomics Bioinformatics. 2024 Mar 23;22(2):qzae028. doi: 10.1093/gpbjnl/qzae028 (PMC12016564; doi:10.1093/gpbjnl/qzae028)
Supplement: qzae028_Supplementary_Data [file qzae028_supplementary_data.zip › figureS2.docx]

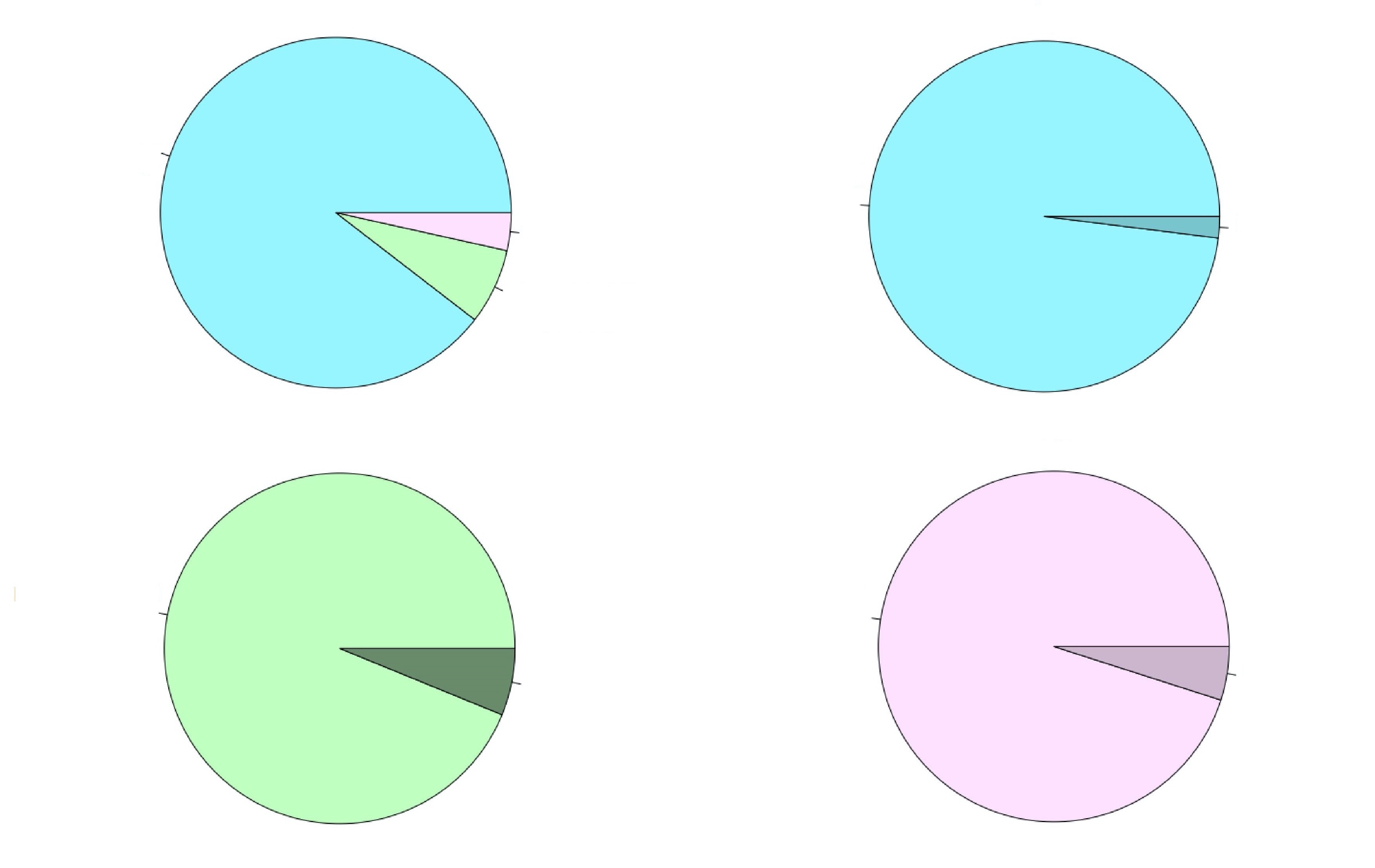


Differential

(1.97%)

**A B**

**C D**

Differential

(4.94%)

Hierarchical-TADs

(6.97%)

Differential

(6.17%)

Non-differential

(95.06%)

Non-differential

(93.83%)

Non-differential

(98.03%)

Complex-TADs

(3.48%)

Single-TADs

(89.55%)
