# Supplementary material for: DiffGR: Detecting Differentially Interacting Genomic Regions from Hi-C Contact Maps
Source: Genomics Proteomics Bioinformatics. 2024 Mar 23;22(2):qzae028. doi: 10.1093/gpbjnl/qzae028 (PMC12016564; doi:10.1093/gpbjnl/qzae028)
Supplement: qzae028_Supplementary_Data [file qzae028_supplementary_data.zip › figureS3.docx]

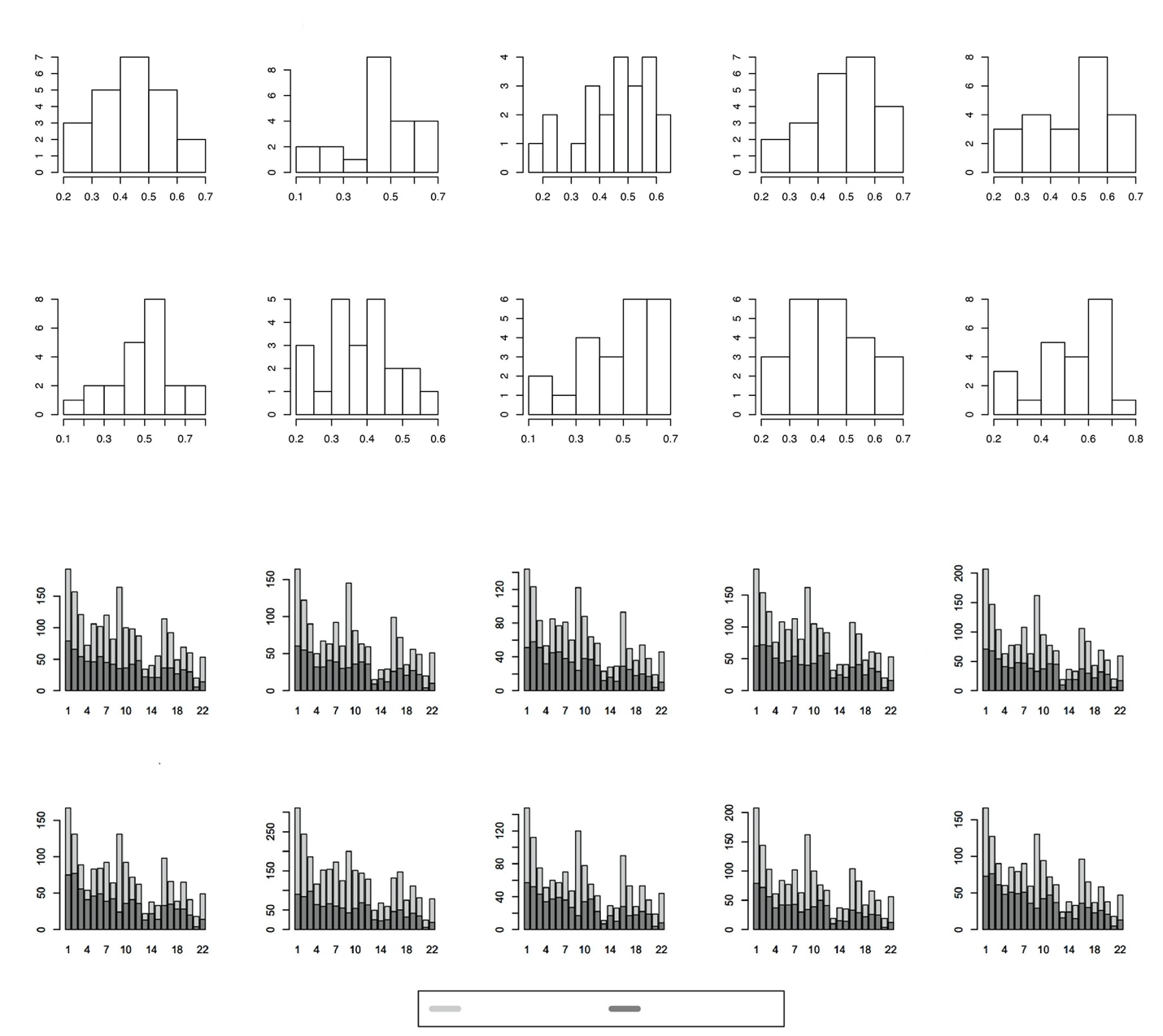


A

B

Candidate regions

Differential regions

Frequency

Frequency

Frequency

Frequency

Frequency

Frequency

Frequency

Frequency

Frequency

Frequency

Frequency

Frequency

Frequency

Frequency

Frequency

Frequency

Frequency

Frequency

Frequency

Frequency

Chromosome

Chromosome

Chromosome

Chromosome

Chromosome

Chromosome

Chromosome

Chromosome

Chromosome

Chromosome

Differential proportion

Differential proportion

Differential proportion

Differential proportion

Differential proportion

Differential proportion

Differential proportion

Differential proportion

Differential proportion

Differential proportion

**K562 *vs* NHEK**

**K562 *vs* NHEK**

**HMEC *vs* K562**

**HMEC *vs* K562**

**HMEC *vs* NHEK**

**HUVEC *vs* NHEK**

**HMEC *vs* NHEK**

**HMEC *vs* NHEK**

**HMEC *vs* K562**

**HMEC *vs* K562**

**HMEC *vs* NHEK**

**HMEC *vs* NHEK**

**GM12878 *vs* NHEK**

**GM12878 *vs* NHEK**

**GM12878 *vs* K562**

**GM12878 *vs* K562**

**GM12878 *vs* HUVEC**

**GM12878 *vs* HUVEC**

**GM12878 *vs* HMEC**

**GM12878 *vs* HMEC**
