# Supplementary figures and images for: DiffGR: Detecting Differentially Interacting Genomic Regions from Hi-C Contact Maps
Source: Genomics Proteomics Bioinformatics. 2024 Mar 23;22(2):qzae028. doi: 10.1093/gpbjnl/qzae028 (PMC12016564; doi:10.1093/gpbjnl/qzae028)

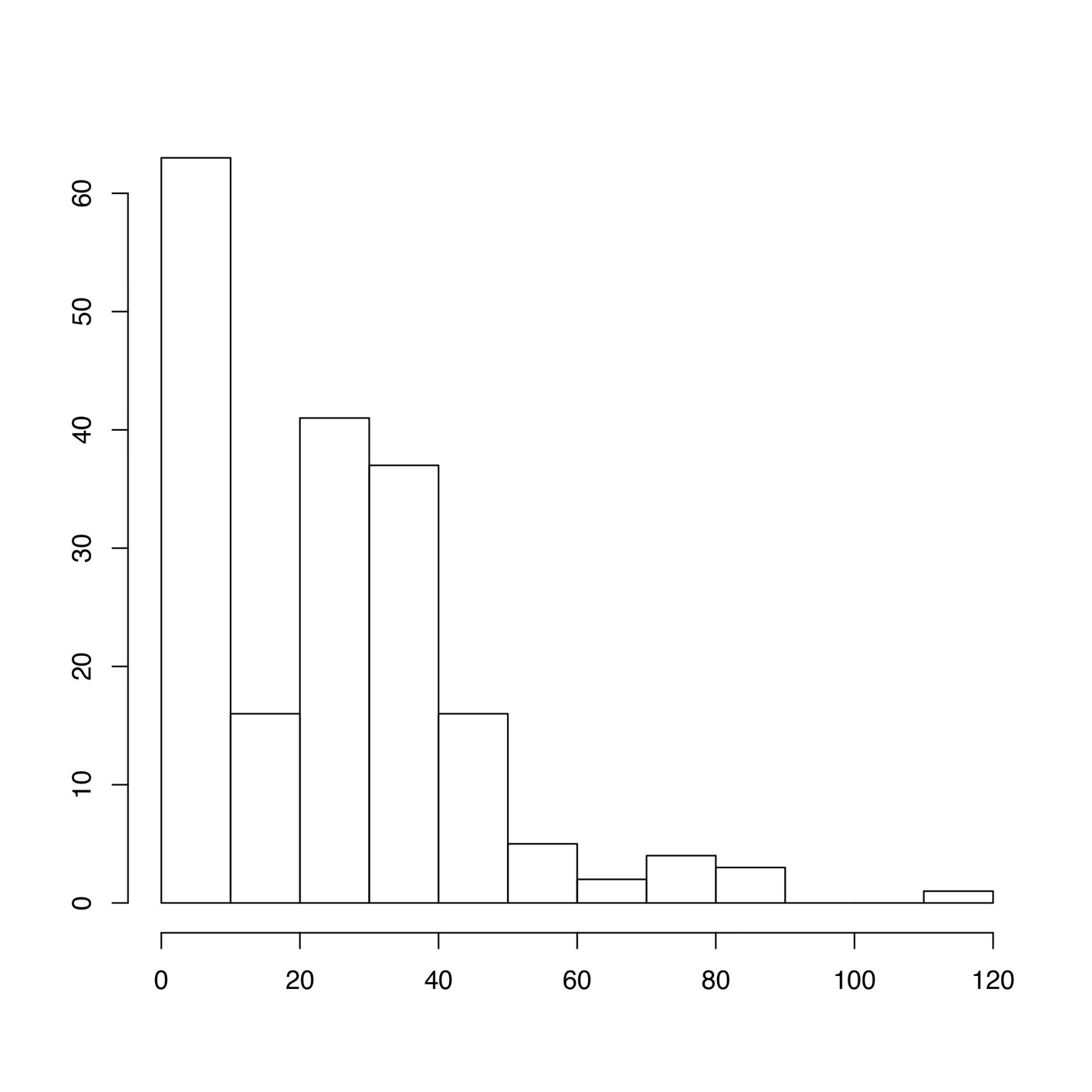


Frequency

TAD size (x 50 kb)

Supplement: qzae028_Supplementary_Data [file qzae028_supplementary_data.zip › figureS1.docx]
